# Ion- and pH-Responsive In Situ Gel Incorporating Luteolin-Loaded Nanostructured Lipid Carriers Enhances Ocular Bioavailability and Anti-Angiogenic Efficacy for Corneal Neovascularization

**DOI:** 10.3390/pharmaceutics18080908

**Published:** 2026-07-24

**Authors:** Yinjian Ji, Zhen Liang, Jingjing Yang, Guojuan Pu, Xue He, Ming Jiang, Tao Wu, Junjie Zhang, Tianyang Zhou, Yuwei Wang

**Affiliations:** 1 Department of the First Clinical Medicine, Henan University of Chinese Medicine, Zhengzhou 450003, China; jyj17856819199@126.com (Y.J.);; 2 Henan Eye Hospital, Henan Provincial People’s Hospital, People’s Hospital of Zhengzhou University, Zhengzhou 450003, China

**Keywords:** luteolin, in situ gel, ion- and pH-responsive, enhance ocular bioavailability, corneal neovascularization

## Abstract

**Background/Objectives:** Corneal neovascularization (CNV) is a leading cause of vision loss, but current treatments are limited by poor ocular drug penetration and rapid tear clearance. Luteolin (LUT) is a poorly water-soluble natural anti-angiogenic agent. To address this limitation, we develop an ion- and pH-responsive in situ gel system (LUT-NLC-ISG) by incorporating LUT-loaded nanostructured lipid carriers (LUT-NLC) into a gellan gum/Carbopol matrix, aiming to enhance ocular bioavailability and therapeutic efficacy against CNV. **Methods:** LUT-NLC-ISG was optimized using a central composite design-response surface methodology (CCD-RSM) and characterized by physicochemical properties (particle size, viscosity, gelation behavior). Ocular pharmacokinetics and biodistribution were evaluated in rabbits after a single topical administration. Biocompatibility was assessed via Hen’s egg test–chorioallantoic membrane assay (HET-CAM), Draize tests, and cytotoxicity studies. Therapeutic efficacy and mechanism were investigated in a murine model of alkali burn-induced CNV. **Results:** The optimized LUT-NLC-ISG had a particle size of 25.27 ± 0.23 nm and exhibited a 45-fold viscosity increase upon simulated tear fluid (STF) exposure. In rabbits, LUT-NLC-ISG significantly increased the bioavailability of LUT in ocular tissues compared with LUT-NLC alone, with 2.57-, 1.83-, and 10.59-fold higher area under the concentration–time curve (AUC) in the cornea, conjunctiva, and tears, respectively and exhibited excellent ocular biocompatibility. In the CNV mouse model, 0.1% (*w*/*v*) LUT-NLC-ISG effectively inhibited corneal neovascularization, comparable to 0.025% dexamethasone, and downregulated VEGF-A and MMP-9 expression. **Conclusions:** LUT-NLC-ISG synergistically combines NLC technology and dual-sensitive in situ gelation to significantly improve LUT ocular bioavailability, offering a promising non-invasive candidate for CNV management.

## 1. Introduction

The cornea is avascular and transparent [1]. Invasion of the cornea by blood or lymphatic vessels is usually associated with inflammatory, infectious, traumatic, immunological, or degenerative disorders of the cornea or ocular surface. Consequently, the corneal tissue loses its transparency, leading to vision loss or blindness. Corneal neovascularization (CNV) is one of the leading causes of visual impairment worldwide, with a global prevalence of 4.1–10.1%. Moreover, 12–57% of affected patients become blind as a consequence [2]. Current CNV treatments primarily involve pharmacological therapy and surgical interventions, but some of these therapies are associated with certain adverse effects. For example, frequent and prolonged use of topical glucocorticoids increases the risk of infection, intraocular hypertension, cataracts, and glaucoma [3]. Anti-angiogenic therapies such as bevacizumab, ranibizumab, and aflibercept, although successful in treating choroidal and retinal neovascularization, have limited efficacy against pathological CNV and are associated with adverse effects, most notably persistent epithelial defects. Furthermore, their high cost restricts clinical application [4,5,6]. Therefore, developing non-invasive, effective, and safer topical therapies for CNV is essential.

Luteolin (LUT) is a polyphenolic flavonoid found in various plants and is recognized for its broad spectrum of bioactive properties. Previous studies have demonstrated that LUT is effective in treating dry eye, cataracts, age-related macular degeneration, and uveitis, as well as in inhibiting retinal neovascularization, glaucoma, and corneal inflammation [7,8,9]. Specifically, LUT exhibits anti-angiogenic activity in retinal diseases [10]. Despite its therapeutic potential, research on LUT in ocular drug delivery remains limited. The poor aqueous solubility of LUT (30 μg/mL in water at 25 °C) limits the concentration of dissolved drug available for corneal absorption [11]. Furthermore, due to tear dilution, the ocular bioavailability of conventional topical eye drops is less than 5% [12]. Moreover, the cornea imposes rigid permeability barriers that hinder the penetration of LUT into the anterior segment [13]. Thus, these obstacles necessitate the development of alternative strategies to improve drug delivery to the eye.

To address the above limitations, nanostructured lipid carriers (NLC) have emerged as a promising strategy. NLC consist of a solid lipid matrix blended with liquid lipids, representing an advanced generation of solid lipid nanoparticles (SLN). Compared with SLN, the inclusion of liquid lipids enhances drug loading capacity, minimizes drug expulsion during storage, and allows modulation of drug release profiles by tailoring the lipid matrix. NLC also improve corneal penetration of drugs, particularly hydrophobic ones [14]. However, the benefits of NLC alone are insufficient to overcome the rapid clearance of topically applied ophthalmic formulations due to tear turnover and nasolacrimal drainage [15]. To overcome this, in situ gelling systems have been developed, which undergo a sol-to-gel transition in response to stimuli (temperature, pH, or ionic strength), thereby enhancing corneal retention [16,17]. The incorporation of NLC into in situ gelling systems further increases the viscosity of the formulation and prolongs ocular residence time [18]. Among the various polymers available for in situ gels, Carbopol (e.g., Carbopol ETD 2020, CB) is widely used in pH-triggered ophthalmic gels due to its low-concentration gelation (0.1–0.5% *w*/*v*), favorable organoleptic properties, viscosity enhancement and mucoadhesive characteristics [17,18,19,20]. However, higher CB concentration, while increasing viscosity, can also lead to excessive acidity and potential ocular irritation. To address this limitation while maintaining gel performance, CB is combined with gellan gum (GG), an ion-activated anionic polymer that gels quickly upon exposure to tear cations (Na^+^, K^+^, Ca^2+^) even at concentrations as low as 0.1% (*w*/*v*) [21]. Moreover, GG has been reported to enhance the thermal stability of CB, maintaining stability under high-temperature conditions [22].

On the basis of this rationale, we developed a novel ion- and pH-responsive in situ gel incorporating LUT-loaded NLC (LUT-NLC-ISG), optimized using a central composite design-response surface methodology (CCD-RSM). This system synergistically combines (1) LUT-NLC to improve corneal penetration and (2) GG and CB to enable ion- and pH-responsive gelation that prolongs ocular residence time, thereby improving ocular bioavailability for effective CNV therapy. In addition to a full physicochemical characterization of LUT-NLC-ISG, this study investigated its ocular biodistribution, corneal penetration capability, safety profiles in vitro and in vivo, and therapeutic outcomes in murine models of CNV.

## 2. Materials and Methods

### 2.1. Materials

Luteolin (LUT) was purchased from Macklin Biochemical Technology Co., Ltd. (Shanghai, China). Monolaurin (Mon) was obtained from Tokyo Chemical Industry Co., Ltd. (Shanghai, China) and J&K Scientific Technology Co., Ltd. (Beijing, China), respectively. Propylene glycol monocaprylate (Capryol^®^ 90, Cap) and PEG-40 hydrogenated castor oil (Cremophor^®^ RH40) were kindly provided by Gattefossé (Saint-Priest, France) and BASF SE (Ludwigshafen, Germany), respectively. PEG400 was purchased from Solarbio Science & Technology Co., Ltd. (Beijing, China), while dexamethasone sodium phosphate eye drops were procured from Huaqing Pharmaceutical Co., Ltd. (Xinxiang, China). The Cell Counting Kit-8 (CCK-8) was obtained from Beyotime Biotech Inc. (Shanghai, China), and enzyme-linked immunosorbent assay (ELISA) kits for mouse matrix metalloproteinase-9 (MMP-9) and vascular endothelial growth factor-A (VEGF-A) were acquired from Elabscience Biotechnology Co., Ltd. (Wuhan, China). HPLC-grade methanol was supplied by Merck (Darmstadt, Germany). All other reagents were of analytical grade, and deionized water was used throughout.

### 2.2. Animals and Cells

New Zealand White rabbits (approximately 2 months old; 2.0–2.5 kg) and male BALB/c mice (6–8 weeks old; 18–22 g) were obtained from Huaxing Experimental Animal Farm (Zhengzhou, China). The animals were free from ophthalmic diseases and housed under controlled conditions (22.0 ± 3.0 °C; 12 h light/dark cycle) with free access to standard chow and water. All animal procedures were approved by the Experimental Animal Ethics Committee of the Henan Institute of Ophthalmology (No.: HNEECA-2023-08), and the experiments were conducted in accordance with the ARVO Statement. Human corneal epithelial (HCE-2) and conjunctival epithelial (CCL-20.2) cell lines were obtained from ATCC (Manassas, VA, USA).

### 2.3. Formulation Screening and Optimization of LUT-NLC and LUT-NLC-ISG

#### 2.3.1. Screening and Optimization of LUT-NLC

Solid lipids, liquid lipids, surfactants, and co-surfactants were screened to identify the excipients with the highest solubility for LUT, and the emulsifying abilities of co-surfactants were compared. Subsequently, solid-to-liquid lipid ratio screening experiments were conducted to determine the optimal solid-to-liquid ratio. Thereafter, pseudo-ternary phase diagrams were constructed to identify the microemulsion region, which provided critical formulation boundaries. Specifically, the microemulsion zone defined the feasible minimum and maximum concentrations of mixed lipid weight (X_1_) and total weight of surfactants and co-surfactants (X_2_) for the subsequent central composite design-response surface methodology (CCD-RSM), with drug loading (DL, Y_1_) and droplet size (DS, Y_2_) as the dependent responses. LUT-loaded nanostructured lipid carriers (LUT-NLC) were prepared using a microemulsion method, in which Mon, Cap, RH40, and PEG400 were gently mixed to form a monophasic mixture under stirring in a water bath at 40 °C. LUT was dissolved in this mixture, followed by the dropwise addition of deionized water under vortexing to achieve a homogeneous microemulsion. The resulting microemulsion was subsequently cooled to room temperature to allow solidification of the lipid matrix, thereby transforming the liquid microemulsion droplets into nanostructured lipid carriers (NLC). The resulting NLC dispersion was adjusted to the final volume and stored at 4 °C for further characterization.

#### 2.3.2. Optimization and Preparation of LUT-NLC-ISG

To optimize the in situ gel formulation, two evaluation indices were used: V_1_ (viscosity at 25 °C) and V_2_ (viscosity at 34 °C after mixing the gel with simulated tear fluid (STF, 25:7 *v*/*v*) [23]. Viscosity was measured at 5 rpm using a viscometer (model DV-II+Pro, Brookfield Engineering Laboratories, Inc., Middleboro, MA, USA) with a CP-51 spindle. STF was composed of NaCl (6.8 g), NaHCO_3_ (2.2 g), CaCl_2_·2H_2_O (0.084 g), and KCl (1.4 g) per liter of deionized water. The ratio of GG to CB was optimized using CCD-RSM. To minimize ocular irritation, the concentration ranges were set at 0.1–0.5% (*w*/*v*) for CB (X_3_) and 0.1–1.0% (*w*/*v*) for GG (X_4_). A two-factor, five-level design was applied (Table S2), with specific compositions determined using Design-Expert 13 software.

For preparation, GG was dispersed in deionized water under continuous stirring at 70–80 °C until completely dissolved. After cooling to room temperature, the required amount of CB was added to the LUT-NLC mixture under constant agitation. The GG solution and the CB-containing LUT-NLC solution were then mixed to obtain a final LUT concentration of 0.1% (*w*/*v*). The formulation (LUT-NLC-ISG) was stored at 4–8 °C.

#### 2.3.3. Preparation of LUT Suspension

A LUT suspension (LUT-Susp) was prepared by dispersing LUT in phosphate-buffered saline containing 0.2% (*w*/*v*) sodium carboxymethyl cellulose, followed by ultrasonication. This suspension served as a control in the in vitro drug release study and other subsequent investigations.

### 2.4. Characterization of LUT-NLC and LUT-NLC-ISG

#### 2.4.1. Morphology Observation of Blank NLC and LUT-NLC

Transmission electron microscopy (TEM, FEI200C, Thermo Fisher, Waltham, MA, USA) was used to observe the morphology of blank NLC and LUT-NLC. A drop of each formulation was placed onto a separate copper grid. After removing the excess solution with filter paper, the grids were negatively stained with 2% (*w*/*v*) uranyl acetate for 1 min. Once dried, the copper grids were loaded into the TEM system for imaging.

#### 2.4.2. Determination of Droplet Size, Polydispersity Index, and Zeta Potential

The average droplet size (DS), polydispersity index (PDI), and zeta potential (ZP) of LUT-NLC and LUT-NLC-ISG were measured using dynamic light scattering (Zetasizer NanoZS90, Malvern Instruments, Worcestershire, UK).

#### 2.4.3. Osmolality and pH Measurement

The pH of the LUT-NLC-ISG formulation was adjusted to approximately 4.0 to ensure stability during storage and tear-triggered gelation upon instillation, while preserving ocular tolerability. The osmolality was determined using a freezing point osmometer (STY-1A, Tianjin, China) and adjusted to 270–325 mOsm/kg by adding appropriate amounts of glycerin, which is within the physiological isotonic range of human tears, to ensure ocular biocompatibility and minimize discomfort upon instillation. Each measurement was performed in triplicate.

#### 2.4.4. Determination of Encapsulation Efficiency and Drug Loading

The amount of LUT loaded into NLC-ISG was calculated by determining the concentration of free drug in the continuous phase. Centrifugal filter units (Amicon^®^ Ultra-4, regenerated cellulose, MWCO 10 kDa; Cork, Ireland) were used to separate unencapsulated LUT by centrifugation (Centrifuge 5810R, Hamburg, Eppendorf, Germany) at 4000 rpm for 10 min at 25 °C. The filtrate was analyzed by HPLC to quantify the unencapsulated LUT. Encapsulation efficiency (EE) and drug loading (DL) were then calculated relative to the initial amount of LUT used in the formulation, according to the following Equations (1) and (2):

(1) $$\begin{array}{c} \mathrm{EE}\left(\%\right)=\frac{W_{a}-W_{f}}{W_{a}}\times 100 \end{array}$$

(2) $$\begin{array}{c} \mathrm{DL}\left(\%\right)=\frac{W_{a}-W_{f}}{W_{a}-W_{f}+W_{L}}\times 100 \end{array}$$

where *W_a_* was the initial amount of the LUT to be added to the LUT-NLC system, *W_f_* was the amount of free LUT in the filtrate, and *W_L_* was the weight of mixed lipid, surfactant and cosurfactant added to the system.

#### 2.4.5. Differential Scanning Calorimetry (DSC)

DSC analysis (TA Instruments, New Castle, DE, USA) was performed to assess the crystalline or amorphous state of LUT in the NLC-ISG formulation. Briefly, blank NLC-ISG and LUT-NLC-ISG were lyophilized using a freeze dryer (CoolSafe Touch 55-9, Allerød, Denmark) at −50 °C for 72 h. For DSC analysis, samples (LUT, freeze-dried blank NLC-ISG, and freeze-dried LUT-NLC-ISG) were placed in sealed aluminum pans. An empty aluminum pan served as the reference. The temperature was ramped from 25 °C to 400 °C at a scanning rate of 10 °C/min under a nitrogen flow of 20 mL/min.

#### 2.4.6. Surface Morphology Analysis by Scanning Electron Microscopy (SEM)

The surface morphology of LUT-NLC-ISG, with or without STF treatment (25:7, *v*/*v*), was examined using scanning electron microscopy (SEM; JSM-6060, JEOL Ltd., Tokyo, Japan). Samples were snap-frozen in liquid nitrogen, freeze-dried for 24 h, and coated with platinum to enhance conductivity. They were then mounted on brass stubs and imaged at an accelerating voltage of 10 kV.

#### 2.4.7. Infrared Spectroscopy Analysis

Infrared spectra were recorded for the following samples: an aqueous solution containing 0.1% LUT, blank NLC-ISG, physical mixture (PM), PM with STF (PM+STF, 25:7 *v*/*v*), LUT-NLC-ISG, and LUT-NLC-ISG+STF. Spectra were collected over the wavenumber range of 800–3000 cm^−1^.

#### 2.4.8. Rheological Evaluation of LUT-NLC-ISG

Rheological analysis was performed using a rotary rheometer (Haake Mars, Thermo Fisher Scientific, Karlsruhe, Germany) equipped with a cone-plate geometry (cone diameter: 35 mm, cone angle: 2°). The shear stress of the formulation was measured over a range of shear rates under two conditions: at 25 ± 0.1 °C without STF and at 34 ± 0.1 °C with STF. A typical run consisted of increasing the shear rate from 0 to 100 s^−1^ at a controlled ramp speed, with a 35 s hold at each shear rate. All measurements were performed in triplicate. The flow behavior of the formulation was determined from the plots of shear stress versus shear rate and viscosity versus shear rate [24].

#### 2.4.9. In Vitro Drug Release

The in vitro release of LUT from LUT-NLC-ISG, LUT-NLC, and LUT-Susp (each containing 1 mg) was evaluated using the dialysis bag method (MWCO 10 kDa) under sink conditions. Each formulation was placed in a dialysis bag and immersed in 100 mL of STF (pH 7.4, containing 0.25% Tween 80 to maintain sink conditions) at 37 °C with shaking at 100 rpm. At 0.5, 1, 2, 4, 6, 8, 12, 24, 36, 48, and 72 h, 0.2 mL samples were withdrawn and replaced with an equal volume of fresh medium. The drug concentration was analyzed by HPLC in triplicate. Cumulative release percentages were calculated using the appropriate Equation (3), and release kinetics were modeled using zero-order, first-order, Higuchi, and Korsmeyer-Peppas equations.

(3) $$\begin{array}{c} Q\left(\%\right)=\frac{C_{n}V+V_{i}\sum_{i=0}^{i=n}C_{i}}{W_{0}}\times 100\%\; \end{array}$$

where *W*_0_ is the initial amount of LUT loaded into the dialysis bag, *C_n_* is the LUT concentration in the released medium at *t_n_*, *t_n_* is sampling at the Nth time, *V* is the total volume of the release medium (100 mL), *V_i_* is the sample volume (0.2 mL) at *t_i_* and *C_i_* is the sample concentration at *t_i_*. The chromatographic conditions were modified as follows: an X Bridge C18 column (150 mm × 3.0 mm, 3.5 μm, XTerra^®^ MS, Waters, Wexford, Ireland) was used with a column temperature of 35 °C. HPLC separation was performed using a mobile phase consisting of methanol and 0.1% phosphoric acid aqueous solution (58:42, *v*/*v*), which was filtered through a 0.45 μm Millipore filter. The flow rate was 0.5 mL/min, detection was performed at a wavelength of 348 nm, and the injection volume was 10 μL.

### 2.5. Short-Term Storage Stability

A comprehensive stability assessment of pharmaceutical formulations is essential to ensure product quality and clinical applicability. In this study, the LUT-NLC-ISG system was evaluated under accelerated and short-term stability conditions at three temperatures (4 °C, 25 °C, and 40 °C) for 4 weeks. Key parameters, including droplet size (DS), polydispersity index (PDI), zeta potential (ZP), and drug concentration (DC), were systematically monitored. Changes in viscosity and pH were also assessed under the same storage conditions. All experiments were performed in triplicate, and data are expressed as mean ± SD.

### 2.6. Ocular Pharmacokinetics in Rabbit Eyes

#### 2.6.1. Rabbits and Treatments

In the single-dose study, 63 male New Zealand White rabbits were randomly assigned to three groups: LUT-NLC-ISG, LUT-NLC, and LUT-Susp (21 rabbits per group). Each group was further evenly and randomly divided into 7 subgroups. A 50 μL dose was administered into the lower conjunctival sac of each rabbit’s eye, and the eyelids were held closed for 15 s to prevent solution loss. Tear fluid samples were collected by placing a pre-weighed sterile filter paper disk (8 mm diameter) beneath the lower eyelid for 30 s at specified time intervals (5, 15, 30, 60, 90, 120, and 180 min). The eyelids remained closed for 15 s after administration. Detailed methods are provided in the Supplementary Materials Figure S1 and S2, Table S1−S3. 

At scheduled time points after administration, the rabbits were euthanized by injection of propofol (7.5 mg/kg) and KCl (100 mg/kg) into the marginal ear vein. The eyes were then rinsed with normal saline. The excised corneas and conjunctivas were rinsed, blotted dry, weighed, and stored at −80 °C until extraction.

#### 2.6.2. Analysis of LUT Levels in Ocular Tissues and Tear Fluids

Tissue samples were treated with 400 μL of methanol for drug extraction. Each tissue was then homogenized for 5 min with a homogenizer (SWE-3D, Servicebio^®^, Wuhan, China). All samples were soaked at 4 °C for 24 h, after which they were vortexed for 1 min and centrifuged at 12,000 rpm for 10 min (MiniSpin^®^ Plus, Eppendorf, Hamburg, Germany). The supernatant was then aliquoted into sample vials for HPLC analysis as described in Section 2.4.9.

To ensure reliable quantification, the bioanalytical method for LUT determination in conjunctival, corneal, and tear fluid samples was validated for specificity, linearity, recovery, precision, accuracy, and stability. The method was successfully applied to measure LUT levels in tissue samples for the pharmacokinetic study. Pharmacokinetic parameters were calculated using DAS 2.1.1 software (Bio Guider Medicinal Technology Co., Ltd., Shanghai, China).

#### 2.6.3. Measurement of Bio-Adhesion Force

The bio-adhesion force was determined using a modified physical balance method [25,26]. An excised rabbit cornea was fixed onto a penicillin vial with the epithelial side facing outward and secured using an aluminum cap and a rubber stopper. The vial containing the cornea was maintained at 37 °C for 5 min. A second vial, prepared identically with another cornea, was inverted and connected to a balance, while the first vial was placed on an adjustable-height platform. The gel premixed with simulated tear fluid (STF; 25:7, *v*/*v*) was applied to the corneal surface of the first vial, and the platform height was adjusted to ensure firm contact between the corneal surfaces of the two vials. After 10 min of contact, the bio-adhesion force was measured as the minimum weight required to separate the two vials, and this value was used in Equation (4). The experiment was repeated three times.

(4) $$\begin{array}{c} Bio-adhesion\;force=\frac{W\times g}{A} \end{array}$$

where *W* is the weight required for detachment (grams), *A* is the area of cornea mucosa exposed (0.785 cm^2^), *g* is the acceleration due to gravity (9.8 m/s^2^).

#### 2.6.4. Ex Vivo Corneal Permeation Study

The ex vivo transcorneal permeation of the developed formulations was evaluated using a well-established method described previously [27]. Three LUT formulations (LUT-NLC-ISG, LUT-NLC, and LUT-Susp) were assessed using Franz diffusion cells. Freshly excised rabbit corneas (approximate available area: 0.694 cm^2^) with an accompanying scleral ring of approximately 2 mm were obtained from New Zealand albino rabbits and mounted onto the diffusion cells. The receptor compartment was filled with 3 mL of STF, while 2 mL of each formulation was placed in the donor compartment. The system was maintained at 34 ± 0.5 °C with a stirring speed of 50 rpm. At predetermined time intervals (15, 30, 60, 90, 120, 180, and 240 min), 200 μL samples were withdrawn from the receptor chamber and immediately replaced with an equal volume of preheated fresh STF. The LUT concentration in the collected samples was analyzed by HPLC as described in Section 2.4.9. All experiments were performed in triplicate.

The cumulative amount of LUT permeated at different time points was calculated using the following Equation (5):

(5) $$\begin{array}{c} Q_{n}=\frac{V_{0}}{A}\left(C_{n}+\frac{V}{V_{0}}\sum_{i=1}^{n-1}C_{i}\right) \end{array}$$

where *V*_0_ was the volume of STF in the receptor chamber (5.0 mL); *A* was the diffusion area (0.694 cm^2^); *V* was the sampling volume (200 μL); *C_n_* was the LUT concentration in the receptor chamber at different intervals; and *C_i_* was the LUT concentration in the receptor chamber before determination. The rate of LUT penetration was determined by the apparent permeability coefficient (*P_app_*) and steady-state flux *(J_ss_*) by the following Equations (6) and (7):

(6) $$\begin{array}{c} P_{app}=\frac{∆Q}{∆t{\times C}_{0}\times 60} \end{array}$$

(7) $$\begin{array}{c} J_{ss}=C_{0}\times P_{app} \end{array}$$

where ∆*Q*/∆*t* was the steady-state slope of the linear plot of the amount of LUT in the receiving chamber vs. time, *C*_0_ was the initial concentration of baicalin in the donor chamber.

At the end of the permeation study, the scleral ring around the cornea was cautiously excised; then the cornea was rinsed with water and excess water was removed using a paper filter. The wet weight (*W_a_*) of each cornea sample was measured. Corneas were dried in an incubator at 70 °C for 12 h to obtain the dry corneal weight (*W_b_*). Corneal hydration level (*HL*) was calculated by Equation (8):

(8) $$\begin{array}{c} HL\;(\%)=\frac{W_{a}-W_{b}}{W_{a}}\times 100\% \end{array}$$

### 2.7. In Vitro and In Vivo Safety Evaluation

#### 2.7.1. Hen’s Egg Test–Chorioallantoic Membrane Assay (HET-CAM)

The ocular irritation potential of LUT-NLC-ISG, LUT-NLC, and LUT-Susp was evaluated using the hen’s egg test on the chorioallantoic membrane (HET-CAM). Fertilized hen eggs were incubated at 37.8 °C and 60% relative humidity for 10 days with regular rotation [28,29]. Eggs weighing 50–60 g were divided into six groups (3 per group): negative control (normal saline), positive control (0.1 M NaOH), blank-ISG, LUT-Susp, LUT-NLC, and LUT-NLC-ISG. 0.5 mL of each test sample was applied onto the chorioallantoic membrane and left in contact for 5 min. The membrane was then rinsed with saline and irritation was scored based on the occurrence of hemorrhage, lysis, or coagulation using the following scale: 0 = none (non-irritant); 1 = isolated (slight); 2 = moderate (moderate); 3 = extensive (severe).

#### 2.7.2. Modified Draize Test

The primary ocular irritation potential of the LUT-NLC-ISG formulation was evaluated using the Draize eye test in New Zealand White rabbits. In a single-dose study, 0.1 mL of the LUT-NLC-ISG eye drops was administered into the conjunctival sac of the left eye, followed by gentle eyelid closure for 15 s. The right eye received 0.1 mL of normal saline as a control. Ocular examinations were performed using a slit-lamp microscope (SLM-9E, Chongqing, China) to assess the ocular surface, including conjunctival redness, discharge, chemosis, and the condition of the cornea and iris, under visible and cobalt blue light at 1, 2, 4, 24, 48, and 72 h post-administration. Irritation scores for each group were calculated as the average of the summed scores from six treated eyes according to the Draize criteria. A score of 0–3 at each time point indicated no irritation, whereas a total ocular irritation index above 4, or a score of 2 or 3 for any individual parameter, was considered indicative of significant irritation.

#### 2.7.3. Cytotoxicity Studies in Human Epithelial Cells of the Cornea and Conjunctiva

The in vitro cytotoxicity of blank-NLC-ISG, LUT-NLC, and LUT-NLC-ISG was evaluated using the CCK-8 assay on human corneal epithelial cells (HCE-2) and human conjunctival epithelial cells (CCL-20.2). HCE-2 and CCL-20.2 were seeded in 96-well plates at a density of 1 × 10^4^ cells per well in 100 μL of DMEM/F12 medium supplemented with 10% fetal bovine serum (FBS) and 1% penicillin-streptomycin. After 24 h of pre-incubation at 37 °C under 5% CO_2_, the cells were treated with 100 μL of medium containing blank-NLC-ISG, LUT-NLC, or LUT-NLC-ISG at concentrations of 2, 4, and 8 μg/mL for 24 h or 48 h. Following treatment, 10 μL of CCK-8 solution was added to each well, and the plates were incubated at 37 °C for 4 h. Absorbance was measured at 450 nm using a microplate reader (PerkinElmer 2104, Shanghai, China). Cell viability was calculated according to the following Formula (9):

(9) $$\begin{array}{c} \mathrm{cell}\;\mathrm{viability}=\frac{{OD}_{sample}-{OD}_{blank}}{{OD}_{control}-{OD}_{blank}}\times 100\% \end{array}$$

### 2.8. In Vivo Anti-CNV Efficacy

#### 2.8.1. Alkali Burn Injury Induced CNV in Mice and Treatments

In this study, CNV was induced in 80 male BALB/c mice using the alkali burn method. Briefly, general anesthesia was achieved by intraperitoneal injection of diazepam (5 mg/kg) and butorphanol tartrate (0.8 mg/kg for analgesia), followed by topical application of 0.5% proparacaine hydrochloride eye drops for local anesthesia. A 2 mm diameter filter paper disk soaked with 2 μL of 1 M NaOH for 1 min was placed on the central cornea for 20 s, after which the cornea was rinsed with physiological saline along the conjunctival sac for 1 min to establish the alkali burn model. At 12 h after injury, all mice received topical ocular administration (5 μL per eye, three times daily for seven days). The mice were randomly divided into five groups: negative control (saline), positive control (0.025% dexamethasone, DEX), and three treatment groups receiving LUT-NLC-ISG at concentrations of 0.1% (high, H), 0.05% (medium, M), and 0.025% (low, L) (*w*/*v*).

#### 2.8.2. Quantification of CNV

CNV development in mice was observed and photographed using a digital camera attached to a slit-lamp microscope on days 1, 3, and 7 after treatment. On day 7, CNV was quantified using a flat-mount-based method [30]. Three mice from each group were deeply anesthetized. After exposing the heart, a perfusion needle was inserted into the left ventricle, and 20 mL of normal saline was administered while the right atrium was incised to allow drainage. Subsequently, 20 mL of carbon black ink was perfused to label the newly formed blood vessels, causing the eyes, ears, and forelimbs to turn dark black. The eyeballs were enucleated and fixed in 4% paraformaldehyde for 24 h. Corneas were then dissected, flattened with four radial incisions, and mounted on slides. Images were captured using a light microscope equipped with a camera (AOSVI^®^, Shenzhen, China), and the CNV areas were quantified using ImageJ software (version 1.41o; National Institutes of Health, Bethesda, MD, USA).

#### 2.8.3. Histopathological Observation

On day 7 post-treatment, three mice per group were euthanized by anesthetic overdose. Eyeballs were collected, and corneas were fixed in 4% paraformaldehyde for 48 h. Corneal sections were prepared using standard paraffin processing and stained with H&E. Images were captured using a Nikon 80i fluorescence microscope (Nikon, Tokyo, Japan).

#### 2.8.4. Enzyme-Linked Immunosorbent Assay (ELISA)

On days 3 and 7 post-administration, five mice from each group were randomly selected and sacrificed. The corneas were excised and immediately frozen at −80 °C for later use. Prior to analysis, the samples were thawed at 4 °C for 30 min, washed with PBS, and homogenized. Tissue lysates were prepared by adding 100 μL of RIPA buffer and incubating on ice for 1.5 h. After centrifugation at 12,000 rpm for 5 min at 4 °C, the supernatant was collected. Total protein concentration was determined using the bicinchoninic acid (BCA) method. The levels of VEGF-A and MMP-9 in the supernatant were measured by ELISA according to the manufacturer’s instructions. Absorbance at 450 nm was read using a microplate reader, and the concentrations of VEGF-A and MMP-9 were calculated based on standard curves.

### 2.9. Statistical Analysis

SPSS 21.0 was used for statistical analysis. Data are mean ± SD. Group comparisons were made by one-way ANOVA, and pairwise comparisons by independent *t*-test. Significance was set at *p* < 0.05.

## 3. Results

### 3.1. Preparation and Optimization of LUT-NLC and LUT-NLC-ISG

**Figure 1:**
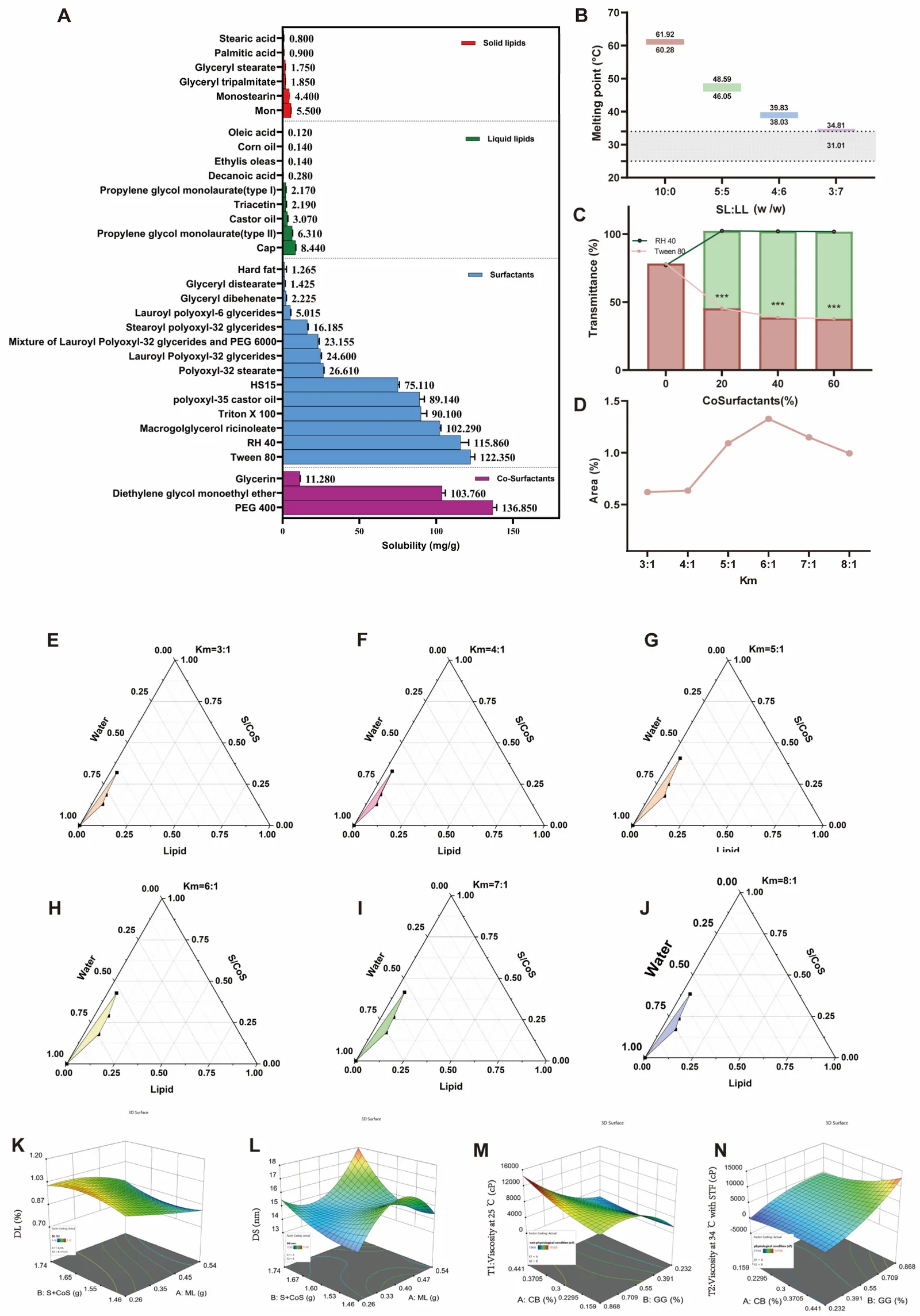
The optimal formulations of LUT-NLC and LUT-NLC ISG were screened as follows: (**A**) Solubility of LUT in solid lipid, liquid lipid, surfactants and cosurfactants (mean ± SD, n = 3). (**B**) The melting point ranges of mixed lipids at different weight ratios of solid lipid (Mon) to liquid lipid (Cap). (**C**) Transmittance (%) of mixed lipid with RH40 and Tween 80 at different ratios of cosurfactant. (RH 40 vs. Tween 80, *** *p* < 0.001). (**D**) Microemulsion areas of different Km values. (**E**–**J**) Pseudoternary phase diagrams consisting of mixed lipids (Mon and Cap), surfactant (RH40) and cosurfactant (PEG400) with different Km values. (**K**,**L**) Three-dimensional response surface charts showing the effect of independent variables on DL and DS. (**M**,**N**) Three-dimensional response surface charts showing the effect of independent variables on V_1_ and V_2_.

To address the dual challenges of poor solubility and limited bioavailability of LUT, this study employed a CCD-RSM approach to optimize the LUT-loaded nanostructured lipid carrier formulation. Based on the solubility of LUT in various excipients (Figure 1A), monostearin (Mon), Capryol^®^ 90 (Cap), RH40, Tween 80, and PEG400 were selected as the solid lipid, liquid lipid, surfactant, and co-surfactant, respectively. As shown in Figure 1B, the optimal solid-to-liquid lipid ratio was determined to be 4:6 by comparing the melting points of mixtures with different ratios. Comparison of the emulsifying ability of different surfactants led to the selection of RH40 due to its superior performance (Figure 1C). The best emulsification effect was achieved at a surfactant-to-co-surfactant ratio of 6:1 (Figure 1D), as confirmed by the pseudo-ternary phase diagrams (Figure 1E–J). Based on these experimental results, the LUT-NLC formulations were optimized using CCD-RSM to achieve desirable DL and DS. Table 1 presents the 13 experimental runs from the design, along with the observed and predicted responses. The mathematical models describing the relationship between the independent variables (X_1_: weight of mixed lipid; X_2_: the total weight of surfactant and co-surfactant) and the dependent variables (Y_1_: DL; Y_2_: DS) are summarized by the following equations:

Y_1_ (DL) = 0.9449 − 0.1213X_1_ + 0.0441X_2_ + 0.0079X_1_X_2_ − 0.0194X_1_^2^ − 0.0004X_2_^2^ − 0.025X_1_^2^X_2_ + 0.0491X_1_X_2_^2^ (R^2^ = 0.95)

Y_2_ (DS) = 14.7 + 0.3359X_1_ − 0.8485X_2_ + 0.46X_1_X_2_ − 0.1666X_1_^2^ + 0.9009X_2_^2^ + 2.14X_1_^2^X_2_ + 0.4341X_1_X_2_^2^ (R^2^ = 0.96)

For DL (Y_1_), the R^2^ value of 0.95 and a *p*-value < 0.05 indicate good model fit (Figure 1K). Both main factors X_1_ and X_2_ had a significant effect on DL (*p* < 0.05). DL was negatively affected by X_1_ (weight of mixed lipid) and positively affected by X_2_ (the total weight of surfactant and co-surfactant). This relationship is further illustrated by the response surface plot. Reducing X_1_ and increasing X_2_ increased drug loading, which can be attributed to the stronger solubilizing ability of the surfactant and co-surfactant for the drug, whereas increasing the lipid content was detrimental to the formation of stable NLC.

For DS (Y_2_), the R^2^ value of 0.96 and a *p*-value < 0.05 also indicate a significant model fit (Figure 1L). Both X_1_ and X_2_ significantly influenced DS (*p* < 0.05). In the core–shell type NLC, the liquid lipid forms the core surrounded by a solid lipid shell. As the lipid content (X_1_) increased, DS initially increased until precipitation occurred, after which DS began to decrease. At low X_2_ levels, the surfactant was insufficient to prevent an increase in DS; at higher X_2_ levels, the particles became encapsulated by excess surfactant, leading to an increase in DS. The optimal formulation was thus determined as follows: Mon 0.357 g, Cap 0.536 g, RH40 3.437 g, and PEG400 0.573 g.

To prolong ocular retention time and further enhance bioavailability, LUT-NLC was incorporated into a composite matrix consisting of CB and GG. Leveraging their dual ion- and pH-sensitive synergistic response, a crosslinked ophthalmic in situ gel (LUT-NLC-ISG) was constructed. To optimize the stability, mechanical properties, and safety of the gel network, the concentrations of CB and GG were re-optimized using CCD-RSM to select formulations that exhibit low viscosity under non-physiological conditions (Figure 1M) and high viscosity under physiological conditions (Figure 1N). According to the optimized CCD-RSM model, the R^2^ values for V_1_ and V_2_ were 0.92 and 0.99, respectively. Both main factors X_3_ (CB concentration) and X_4_ (GG concentration) had extremely significant effects on both responses (*p* < 0.05). The mathematical relationships between each factor and the response values were further clarified through response surface plots. The final optimal in situ gel formulation consisted of 0.322% (*w*/*v*) CB and 0.232% (*w*/*v*) GG dissolved in double-distilled water (Table 2). The fitting results for V_1_ and V_2_ are shown below, indicating good model fit:

V_1_ = 6503.00 − 1186.17X_3_ + 4556.24X_4_ + 1432.6X_3_X_4_ + 783.55X_3_^2^ − 639.45X_4_^2^ + 197.06X_3_^2^X_4_ + 2905.57X_3_X_4_^2^, R^2^ = 0.92

V_2_ = 4075.8 + 655.49X_3_ + 2711.9X_4_ + 1459.65X_3_X_4_ + 533.91X_3_^2^ + 191.81X_4_^2^ + 1281.95X_3_^2^X_4_ + 1808.86X_3_X_4_^2^. R^2^ = 0.99

### 3.2. Characterization of LUT-NLC and LUT-NLC-ISG

**Figure 2:**
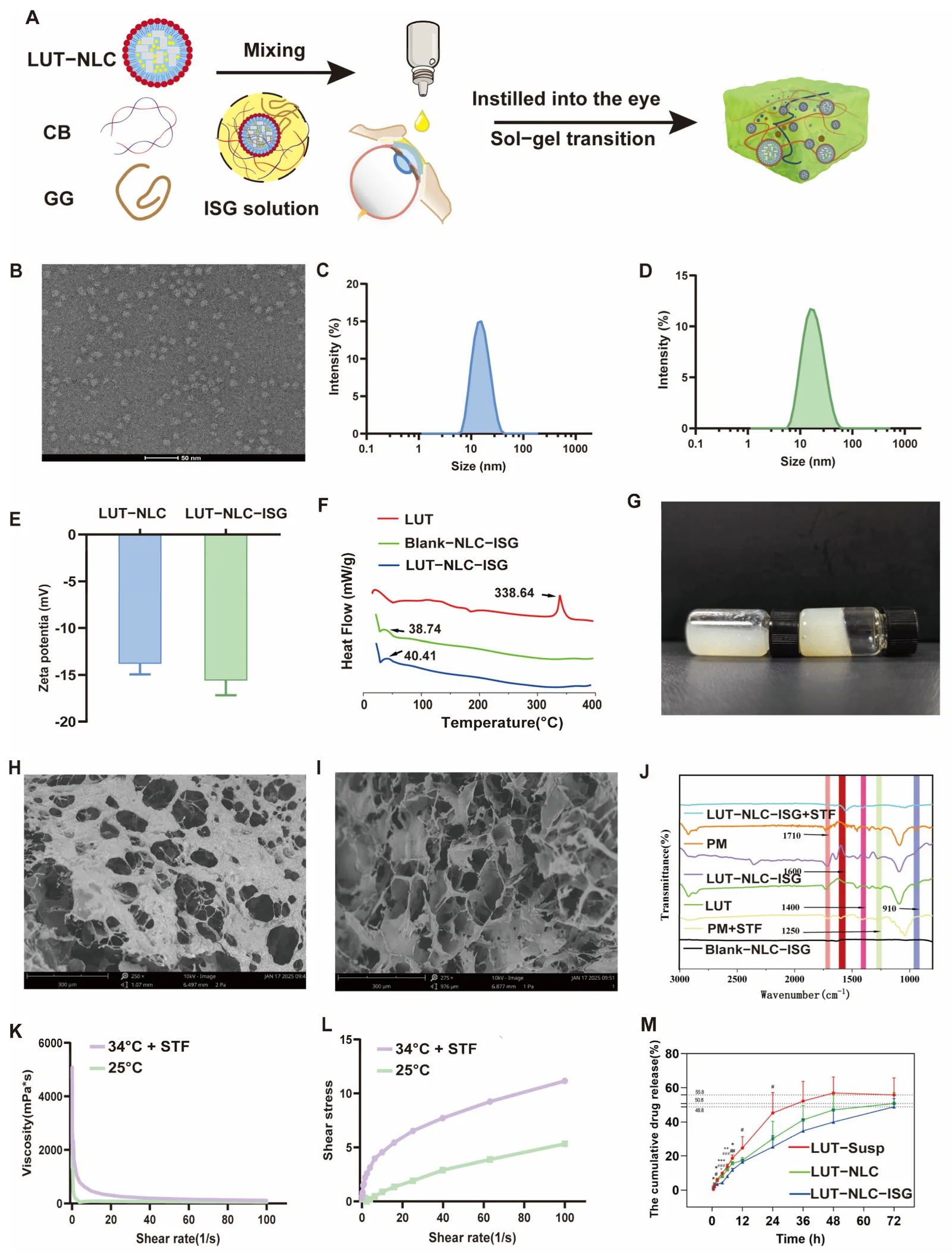
Physicochemical characterization of LUT-NLC and LUT-NLC-ISG: (**A**) Schematic of the formulation preparation. (**B**) TEM images of LUT-NLC, scale bar = 50 nm. (**C**) Size distribution of LUT-NLC. (**D**) Size distribution of LUT-NLC-ISG. (**E**) Zeta potential of LUT-NLC and LUT-NLC-ISG. (**F**) DSC diagrams of the LUT, blank NLC-ISG and LUT-NLC-ISG. (**G**) Appearance of LUT-NLC-ISG. (**H**,**I**) SEM images of the surface morphology of LUT-NLC-ISG without STF and with STF. (**J**) Infrared spectra of LUT-NLC-ISG + STF, PM, LUT-NLC-ISG, LUT, PM + STF, blank-ISG + STF. (**K**,**L**) Rheological evaluation of LUT-NLC-ISG and LUT-NLC-ISG + STF. (**M**) Cumulative in vitro drug release profiles from LUT-NLC-ISG, LUT-NLC and LUT Susp (n = 3, mean ± SD, LUT-NLC-ISG: LUT-NLC: * *p* < 0.05, ** *p* < 0.01, *** *p* <0.001. LUT-NLC-ISG: LUT-Susp: ^#^ *p* < 0.05, ^##^ *p* < 0.01, ^###^ *p* < 0.001; LUT-NLC: LUT-Susp: ^+^ *p* < 0.05).

A schematic of the formulation preparation is illustrated in Figure 2A. LUT-NLC exhibited a clear and uniform appearance. The drug concentration of the prepared LUT-NLC-ISG was approximately 1.00 ± 0.01 mg/mL, which was 33 times higher than its intrinsic aqueous solubility. TEM images showed spherical, uniform droplets for LUT-NLC (Figure 2B). The particle size distribution histogram, obtained by measuring over 100 randomly selected particles from the TEM image using ImageJ software (version 1.41o, National Institutes of Health, Bethesda, MD, USA), is shown in Figure S5. The average particle diameter was determined to be 20.33 nm, which is consistent with the DS distribution. Measurements showed that the particle size of LUT-NLC was about 6 nm larger than that of the blank NLC, indicating that the drug was successfully encapsulated by NLC. The particle size of LUT-NLC was 20.27 ± 0.13 nm (Figure 2C) with a PDI of 0.12 ± 0.03. This size was smaller than the corneal intercellular gap width (20–50 nm), which may facilitate paracellular transport. However, corneal permeation is likely attributable to a combination of factors, including particle size, surface charge, and mucoadhesion, rather than size alone [31]. After assembly of LUT-NLC with ISG to form LUT-NLC-ISG, the particle size increased to 25.27 ± 0.23 nm (Figure 2D) with a PDI of 0.24 ± 0.04, indicating partial aggregation of NLC or polymer adsorption, while still maintaining a narrow and uniform size distribution. After the addition of CB and GG, the zeta potential of LUT-NLC changed from −13.8 ± 1.1 mV to −15.6 ± 1.6 mV for LUT-NLC-ISG (Figure 2E), which was attributed to the negative charges of CB and GG that increased the overall negative potential and enhanced the stability of the final formulation [32]. Benefiting from the preceding formulation screening, the optimized LUT-NLC-ISG exhibited a high encapsulation efficiency of 98.78% ± 0.11%, along with a final formulation pH of 4.19 ± 0.02 and an osmolality of 299.33 ± 1.25 mOsm/kg, complying with the safety and design requirements for eye drops.

DSC thermograms (Figure 2F) were recorded for LUT, blank NLC-ISG and LUT-NLC-ISG lyophilized powder. The DSC analysis of LUT displayed a distinct endothermic peak at approximately 338 °C, which was absent in the thermograms of both blank NLC-ISG and LUT-NLC-ISG, suggesting the incorporation of LUT into the NLC-ISG matrix.

The ISG mechanism involves a stimuli-responsive phase transition, in which the instilled solution forms a gel in the ocular cul-de-sac upon exposure to specific environmental triggers [33]. The addition of simulated tear fluid (STF) at 34 °C to the ISG mimicked the ocular surface environment. LUT-NLC-ISG was liquid at 25 °C. After STF addition, the pH increased to 4.67 ± 0.03, and in the presence of cations from STF, the formulation rapidly formed a gel (Figure 2G). Scanning electron microscopy (SEM) revealed changes in surface morphology upon STF addition. Without STF (Figure 2H), LUT-NLC-ISG appeared loose, with thin, fragile pore walls and fractures. In contrast, with STF (Figure 2I), the gel exhibited a more coherent surface and fewer fractures, confirming the successful formation of a cross-linked in situ gel structure.

Infrared spectroscopy analysis (Figure 2J) was employed to characterize the crosslinking reaction. The study revealed that in the absence of STF, the -COOH characteristic peak at 1710 cm^−1^ persisted in the LUT powder, PM, and LUC-NLC-ISG group. Upon the addition of STF, the LUC-NLC-ISG system exhibited significant changes: the -COOH peak at 1710 cm^−1^ disappeared, while weak symmetric stretching vibration peaks of -COO^−^ emerged in the 1400 cm^−1^ and 1600 cm^−1^ regions. Notably, residual -COOH peaks remained in the PM + STF group, likely due to spectral interference from the complex structure of LUT, though a -COO^−^ absorption peak at 1400 cm^−1^ was still observed. These spectral findings confirm that under the alkaline environment provided by STF, the -COOH in LUC-NLC-ISG underwent acid-base neutralization, successfully converting to -COO^−^. Following the addition of STF to LUT-NLC-ISG, the -OH peak at 1250 cm^−1^ vanished entirely. The findings suggested that crosslinking among LUT-NLC-ISG components occurred via multiple hydrogen bonds between -OH and -COO^−^ groups, resulting in a polymeric network hydrogel. Additionally, near 910 cm^−1^, both the physical mixture and LUT displayed characteristic phenolic hydroxyl peaks of LUT, whereas these peaks were absent in the LUT-NLC-ISG group, indicating possible interactions with the lipid/surfactant matrix, which supports the incorporation of LUT into NLC-ISG [34].

Rheological evaluation of LUT-NLC-ISG, including shear stress and viscosity versus shear rate, was performed. As shown in Figure 2K and the Supplementary Materials Figure S3 and Table S4 and S5, LUT-NLC-ISG exhibited a significant increase in viscosity when exposed to STF at 34 °C, with a 45-fold increase observed at a shear rate of 0.1 s^−1^, compared to the same formulation at 25 °C without STF. Importantly, under STF-induced gelation conditions, the viscosity decreased with increasing shear rate, indicating shear-thinning behavior characteristic of non-Newtonian fluids. This behavior is attributed to the CB/GG polymer network, which undergoes reversible structural disruption under shear, thereby reducing flow resistance during blinking and facilitating uniform spreading of the formulation across the corneal surface. Conversely, under non-gelation conditions (25 °C, without STF), the formulation exhibited Newtonian flow behavior, as evidenced by the linear relationship between shear stress and shear rate (R^2^ = 0.98, Figure 2L). The low initial viscosity under these conditions ensures excellent fluidity and instillation comfort prior to gelation. Collectively, these rheological properties highlight the formulation’s potential for effective ocular drug delivery.

As shown in Figure 2M, LUT-NLC-ISG exhibited a markedly different release profile from LUT-NLC and LUT-Susp. LUT-Susp exhibited the fastest release, with 18.7%, 47.6% and approximately 56% of LUT released at 8 h, 24 h and 72 h, respectively, indicating its limited sustained-release capacity. In contrast, LUT-NLC showed a substantially slower release, with 30.2% at 24 h and 50.8% at 72 h. Notably, LUT-NLC-ISG demonstrated the most pronounced sustained-release behavior, releasing only 2.3% within the first hour, followed by a gradual and controlled release of 25.3% at 24 h and 48.8% at 72 h. The slower release of ISG compared to NLC is attributed to the diffusion restriction imposed by the network structure of the gel [35], which may restrict the mobility of both the NLC particles and the released drug molecules. The release data were further fitted to various kinetic models. As shown in Table 3 and the Supplementary Materials Figure S4, the in vitro release of LUT from the LUT-NLC-ISG, LUT-NLC, and LUT-Susp best fits the first-order kinetic model with the highest correlation, indicating a concentration-dependent release process primarily governed by passive diffusion across the lipid matrix and the aqueous boundary layer [36]. This kinetic model implies that the rate of release is proportional to the concentration of LUT remaining in the formulation, which can be advantageous for maintaining therapeutic levels over an extended period [37]. Collectively, these findings confirm that the NLC-ISG formulation provides superior sustained-release performance, leveraging the colloidal carriers to achieve prolonged and controlled drug delivery, offering a promising strategy for prolonged ocular drug delivery.

### 3.3. Short-Term Storage Stability of LUT-NLC-ISG

**Figure 3:**
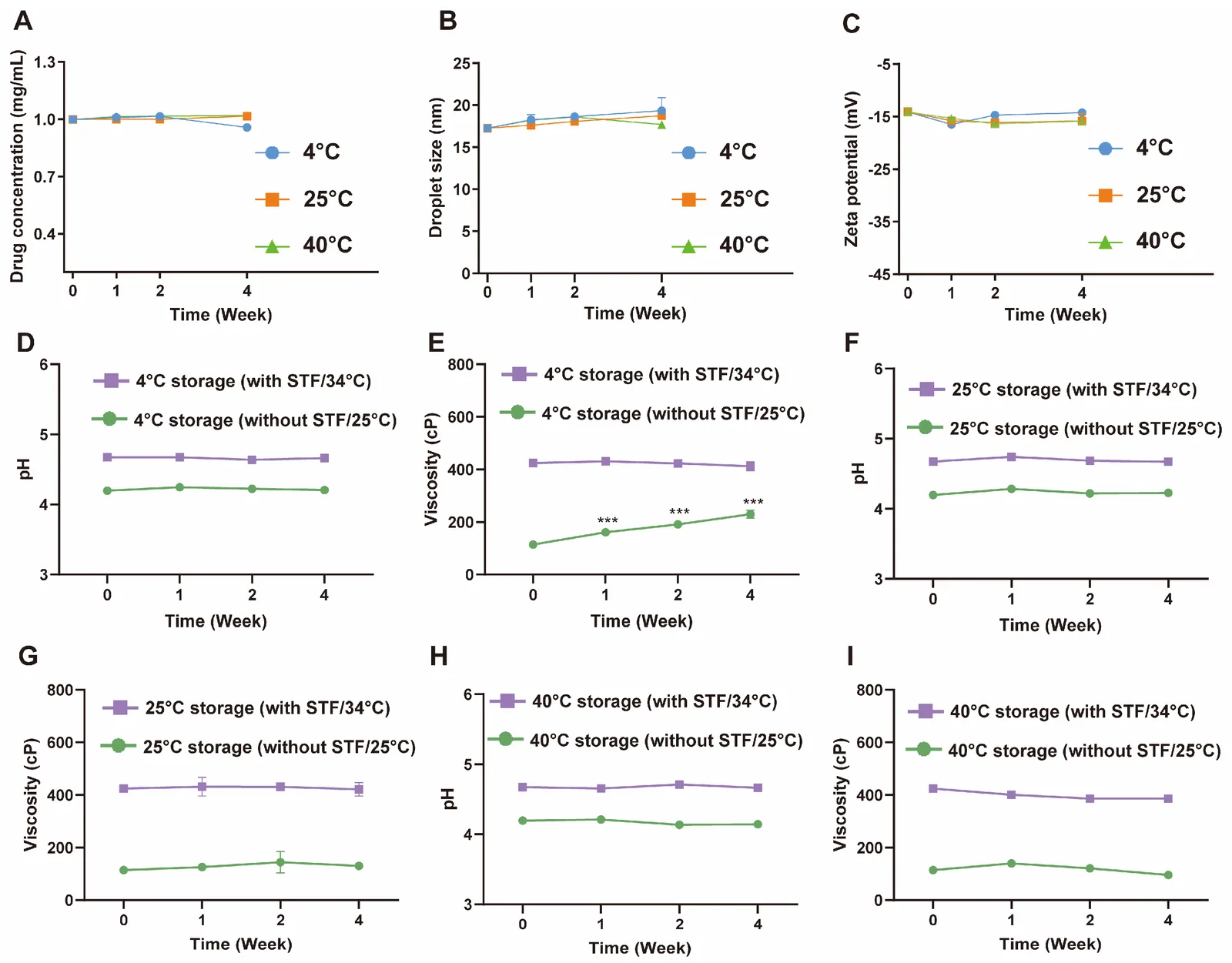
Short-Term Storage Stability of LUT-NLC-ISG. The changes in LUT-NLC-ISG formulation in (**A**) drug concentration, (**B**) droplet size, and (**C**) zeta potential under different conditions. pH and viscosity changes in LUT-NLC-ISG stored at (**D**,**E**) 4 °C, (**F**,**G**) 25 °C, and (**H**,**I**) 40 °C. *** *p* < 0.001.

The short-term stability of LUT-NLC-ISG was evaluated in different storage conditions (4 °C, 25 °C, and 40 °C) over a 4-week duration. As illustrated in Figure 3A–C, no significant changes in drug content (DC), droplet size (DS), and zeta potential (ZP) were observed across any of the storage conditions throughout the evaluation period. Figure 3D–I further illustrate the changes in pH and viscosity of LUT-NLC-ISG under two determination conditions (25 °C without STF and 34 °C with STF) after storage at 4 °C, 25 °C, and 40 °C. The LUT-NLC-ISG samples stored at 40 °C and 25 °C preserved their physicochemical properties without significant changes, indicating sufficient stability under ambient and stressed conditions over the 4-week duration. The pH value of all samples remained within the physiologically acceptable range throughout the study. Overall, these findings demonstrate that LUT-NLC-ISG exhibits satisfactory short-term stability for at least four weeks when stored at 25 °C or even 40 °C. It should be made clear that the 4-week stability data serve as a preliminary assessment, and that long-term stability evaluations (3–6 months) are planned as part of our future preclinical development program.

### 3.4. Ocular Pharmacokinetics Studies of LUT-NLC and LUT-NLC-ISG

**Figure 4:**
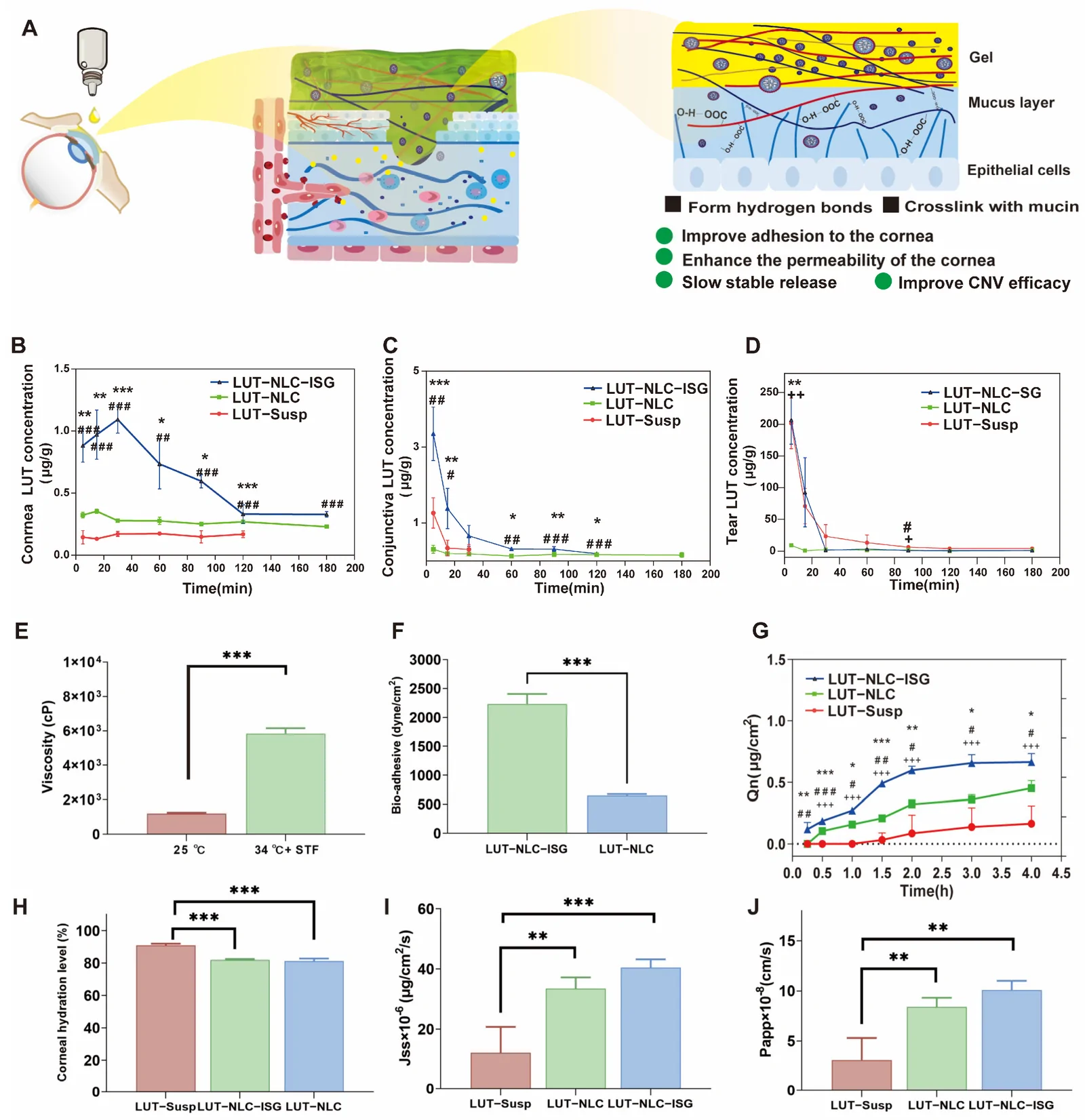
**Ocular pharmacokinetics studies of LUT-NLC and LUT-NLC-ISG.** (**A**) Schematic of the ocular drug delivery of LUT-NLC-ISG. Concentration–time profiles of LUT concentration in cornea (**B**), conjunctiva (**C**), and tear (**D**), n = 6, mean ± SD. (LUT-NLC-ISG: LUT-NLC: * *p* < 0.05, ** *p* < 0.01, *** *p* <0.001. LUT-NLC-ISG: LUT-Susp: ^#^ *p* < 0.05, ^##^ *p* < 0.01, ^###^ *p* < 0.001; LUT-NLC: LUT-Susp: ^+^ *p* < 0.05, ^++^ *p* < 0.01, ^+++^ *p* < 0.001). (**E**) Viscosity of LUT-NLC-ISG without and with STF. (**F**) The Bio-adhesive of LUT-NLC and LUT-NLC-ISG. (**G**) Ex vivo corneal permeation study of LUT-NLC-ISG, LUT-NLC and LUT-Susp (n = 3, mean ± SD, LUT-NLC-ISG: LUT-NLC: * *p* < 0.05, ** *p* < 0.01, *** *p* < 0.001. LUT-NLC-ISG: LUT-Susp: ^#^ *p* < 0.05, ^##^ *p* < 0.01, ^###^ *p* < 0.001; LUT-NLC: LUT-Susp: ^+^ *p* < 0.05, ^++^ *p* < 0.01, ^+++^ *p* < 0.001). (**H**) Corneal hydration, (**I**) J_SS_, (**J**) P_app_ of LUT-NLC-ISG, LUT-NLC, and LUT Susp (n = 3, mean ± SD), ** *p* < 0.01, *** *p* < 0.001.

The ocular pharmacokinetics of LUT were assessed following a single topical administration of LUT-NLC-ISG, LUT-NLC, and LUT-Susp (Figure 4A). Figure 4B–D present the concentration–time profiles of LUT in the cornea, conjunctiva, and tears. In the cornea, the administration of LUT-NLC-ISG resulted in significantly higher LUT concentrations compared to both LUT-NLC and LUT-Susp at all time points (Figure 4B). Specifically, relative to LUT-NLC, LUT-NLC-ISG achieved increases of 2.7-, 2.7-, 3.8-, 2.0-, 1.8-, and 1.1-fold at 5, 15, 30, 60, 90, and 120 min, respectively; the corresponding increases in comparison to LUT-Susp were 9.1-, 7.4-, 6.2-, 3.6-, 4.0-, and 1.9-fold. Similarly, in the conjunctiva (Figure 4D), LUT-NLC-ISG administration resulted in significantly higher LUT concentrations than LUT-NLC at 5, 15, 60, 90, and 120 min, with increases of 9.4-, 7.2-, 2.4-, and 2.2-fold, respectively; the corresponding increases relative to LUT-Susp were 9.1-, 7.4-, 3.6-, and 4.0-fold, respectively. As presented in Table 4, the area under the concentration–time curve (AUC) over 180 min demonstrated that, compared to LUT-NLC, LUT-NLC-ISG increased ocular bioavailability by 2.17-, 2.57-, and 10.62-fold in cornea, conjunctiva, and tears, respectively. When compared to LUT-Susp, the increases are 4.28-, 3.21-, and 0.55-fold, respectively. It is noteworthy that although LUT-Susp exhibited a high AUC in tears, its AUC in cornea and conjunctiva remained significantly lower, indicating that elevated tear concentrations do not necessarily correlate with enhanced tissue penetration. The concentration observed is attributed to the accumulation of large particles on the conjunctiva. However, its overall bioavailability remains suboptimal. In contrast, the high tear concentration observed with LUT-NLC-ISG is attributed to gel formation, which prolongs retention on the ocular surface. The superior corneal concentration of LUT-NLC-ISG compared to both LUT-NLC and LUT-Susp could be partially attributed to the high viscosity of the in situ gel, which provides protection against tear elimination. The viscosity of LUT-NLC-ISG (Figure 4E) increased significantly from 1201.7 ± 51.0 cP at 25 °C without STF to 5849.0 ± 305.1 cP at 34 °C with STF (0.2 rpm). Furthermore, the mucoadhesive force of LUT-NLC-ISG (2231.7 ± 176.8 dyne/cm^2^, Figure 4F) was significantly higher than that of LUT-NLC (651.4 ± 23.8 dyne/cm^2^) and exceeded the tear film shear force (150 dyne/cm^2^) [21], facilitating adherence to the ocular surface. In summary, the gel-induced precorneal retention, enhanced mucoadhesion, and superior corneal penetration of the NLC system synergistically contributed to the significantly improved ocular bioavailability of LUT from the LUT-NLC-ISG formulation.

Corneal permeability is a key determinant of pharmaceutical efficacy and a major factor influencing the bioavailability of ophthalmic formulations. As shown in Figure 4G, cumulative drug permeation increased over time. At the initial time point (0.25 h), the LUT-NLC-ISG group exhibited higher cumulative permeation than the LUT-Susp group but lower than the LUT-NLC group. This is because the gel formed by the LUT-NLC-ISG formulation in the apparatus created a network that hindered LUT diffusion. Nevertheless, the advantages of NLC enabled the LUT-NLC-ISG formulation to still achieve superior permeation over LUT-Susp at 0.25 h. As shown in Figure 4H, the HL values of both the LUT-NLC-ISG and LUT-NLC groups remained below 83%, indicating no damage to corneal epithelial or endothelial cells during the experiment. In contrast, the HL value of the LUT-Susp group reached 90.86 ± 1.11%. The steady-state flux (J_ss_) and apparent permeability coefficient (P_app_) values (Figure 4I,J) of the LUT-NLC-ISG and LUT-NLC groups were significantly higher than those of the LUT-Susp group (*p* < 0.001), indicating that LUT-NLC-ISG significantly enhances transmembrane transport efficiency, facilitating the attainment of therapeutic concentrations in the target tissue. This implies better absorption, potentially higher bioavailability, and improved therapeutic outcomes [38].

### 3.5. In Vitro and In Vivo Safety Evaluation

**Figure 5:**
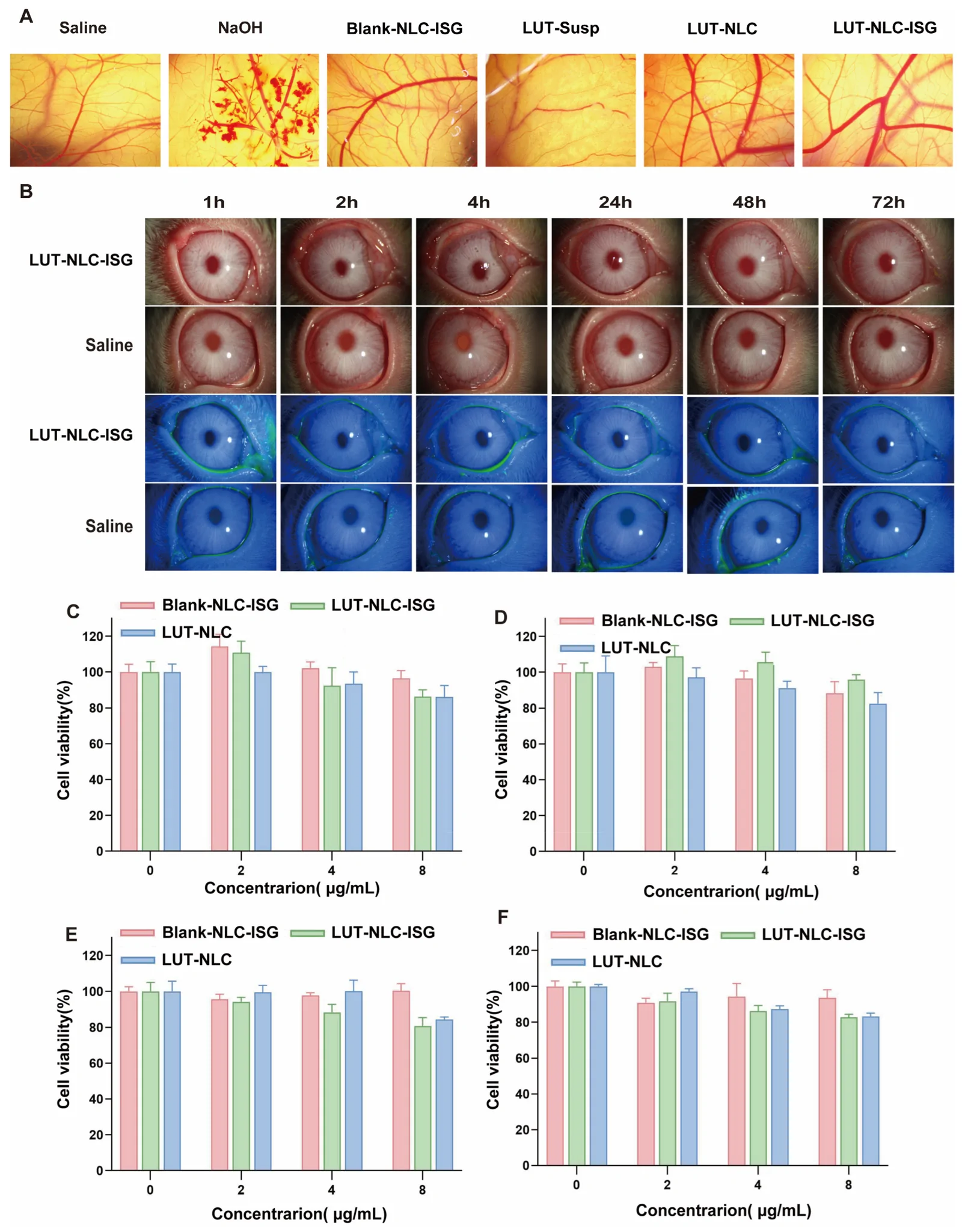
In vitro and in vivo safety evaluation. (**A**) Irritant effects of substances applied to the CAMs over a 5 min period (n = 3); (**B**) images of rabbit eyes observed by slit-lamp microscope after one application of LUT-NLC-ISG and saline (as control) at different time points under visible light and cobalt blue light after being stained with fluorescein sodium. Cell activity of blank-NLC-ISG, LUT-NLC, and LUT-NLC-ISG; (**C**) CCL-20.2 activity at 24 h; (**D**) CCL-20.2 activity at 48 h; (**E**) HCE-2 activity at 24 h; (**F**) HCE-2 activity at 48 h; n = 6, mean ± SD).

The HET-CAM test was employed to assess the acute irritancy potential of the formulations. As shown in Figure 5A, the positive control (0.1M NaOH) induced marked hemorrhage and vascular lysis, whereas the negative control (normal saline) showed no visible irritation. Similarly, treatment with blank-NLC-ISG, LUT-Susp, LUT-NLC, and LUT-NLC-ISG did not induce a detectable hemorrhage, vascular lysis, or coagulation on the CAM vessels within the 5 min observation period. These results demonstrated that neither LUT, the NLC-ISG carrier, nor the LUT-loaded formulation exhibited irritant effects under the tested conditions.

Ocular irritation was assessed by slit-lamp microscopy after topical administration of LUT-NLC-ISG (Figure 5B). Over three consecutive days, neither the formulation-treated group nor the saline group showed ocular damage. Additionally, sodium fluorescein staining under cobalt blue light confirmed an intact corneal epithelium without defects. These results highlight the biocompatibility of LUT-NLC-ISG.

The cytotoxicity of LUT-NLC-ISG and LUT-NLC against HCE-2 and CCL-20.2 was determined by the CCK-8 cell viability assay. As shown in Figure 4C–F, LUT-NLC-ISG and LUT-NLC induced a concentration-dependent reduction in cell viability. The cell viability of HCE-2 and CCL-20.2 treated with 8 μg/mL LUT-NLC-ISG remained above 80% at both 24 h and 48 h (Figure 5C–F), indicating acceptable biocompatibility of the formulations. Collectively, these findings support the safety and therapeutic potential of LUT-NLC-ISG and LUT-NLC for CNV treatment at the tested concentrations.

### 3.6. In Vivo Anti-CNV Efficacy

**Figure 6:**
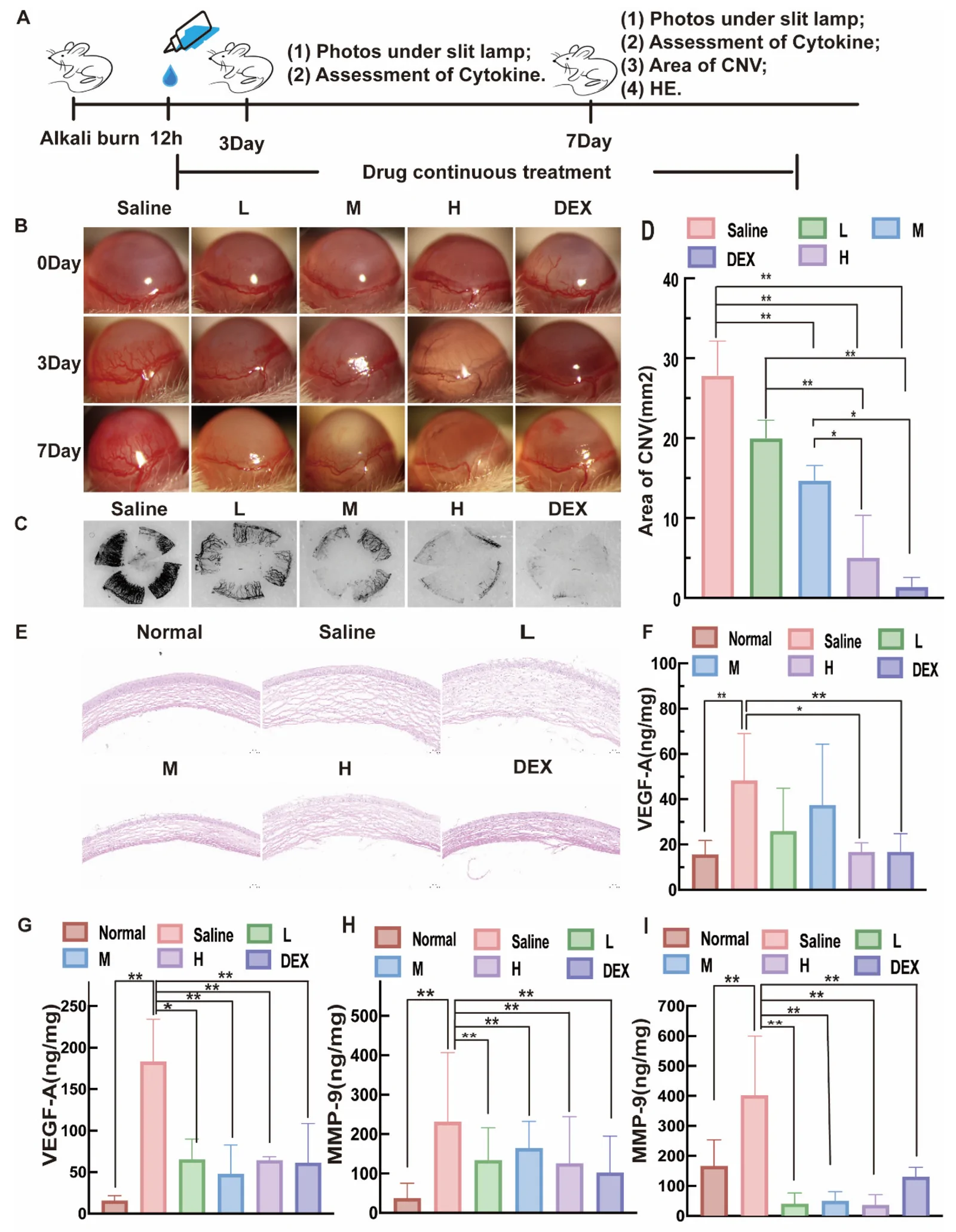
In vivo anti-CNV efficacy of LUT-NLC-ISG. (**A**) Illustration of the construction of CNV model and the therapeutic profile. (**B**) Representative images of the CNV on days 0, 3, and 7 after treatment. (**C**) Representative images of corneal flat mounts are displayed under each group. (**D**) The area of CNV in the five groups, * *p* < 0.05, ** *p* < 0.01. (**E**) Histopathological examination of mice cornea (300×). (**F**,**G**) Protein expression of VEGF-A in the cornea at day 3 and day 7 post-alkali burn. (**H**,**I**) Protein expression of MMP-9 in the cornea at day 3 and day 7 post-alkali burn (n = 5, mean ± SD, compared to saline: * *p* < 0.05, ** *p* < 0.01).

To evaluate the antiangiogenic activity of optimized LUT-NLC-ISG, an in vivo CNV assay was performed using an alkali burn-induced mouse model (Figure 6A). After corneal injury, mice were observed and randomly assigned to five groups. Corneal neovascularization was monitored by slit lamp microscope on days 1, 3, and 7 (Figure 6B). The saline group showed progressive time-dependent vascular proliferation: on day 3, neovascularization increased significantly, and on day 7, dense neovessels had covered almost the entire cornea. In contrast, all treatment groups (L, M, H, and DEX groups) showed only a slight increase in neovascularization on day 3. By day 7, the L and M groups still exhibited a small amount of neovascularization, which was less extensive than that observed on day 3, whereas the H and DEX groups showed almost complete disappearance of neovascularization.

Quantitative analysis (Figure 6C) revealed that LUT-NLC-ISG significantly reduced the CNV area in a dose-dependent manner after seven days of treatment. M, H, and DEX groups showed significantly reduced CNV area compared with the saline groups (*p* < 0.01, Figure 6D). Both H and DEX groups exhibited significantly greater anti-CNV efficacy than the L group (*p* < 0.01) and the M group (*p* < 0.05), with no significant difference between the H and the DEX groups (*p* > 0.05).

Histopathological evaluation was performed by H&E staining to further assess the effects of different treatments on alkali burn-induced corneal damage. As shown in Figure 6E, the normal group displayed a well-organized epithelial layer and orderly stromal collagen, with a uniform and clear overall structure. Conversely, the saline-treated group presented with corneal edema, inflammatory cell infiltration in the stroma and loosely arranged collagen fibers with intervening gaps, resulting in increased stromal thickness. The corneal epithelium in the saline group was disorganized with irregularly arranged cells. However, the LUT-NLC-ISG group showed a substantial reduction in infiltrated cells and stromal thickness. Corneas from the H and DEX groups displayed pronounced structural restoration and reduced neovascularization throughout the tissue. These findings aligned with the quantitative CNV area measurement and further support the anti-angiogenic efficacy of high-dose LUT-NLC-ISG therapy.

To explore the anti-neovascularization mechanism of LUT, corneal levels of VEGF-A and MMP-9 were measured using ELISA (Figure 6F–I) on days 3 and 7. As shown in Figure 6F–I, VEGF-A and MMP-9 levels in corneal tissues were significantly reduced in LUT-NLC-ISG treatment (H) and DEX groups compared to the saline group on days 3 and 7 post-treatment (*p* < 0.05). No significant differences among the three LUT-NLC-ISG treatment groups (L, M, and H) (*p* > 0.05). The expression levels of both protein markers displayed no significant difference between H and DEX groups (*p* > 0.05), suggesting that LUT-NLC-ISG (H group, 0.1%) exerts comparable anti-CNV activity to that of dexamethasone under the tested conditions, over the 7-day treatment period.

## 4. Discussion

Corneal transparency is essential for clear vision, and the avascular nature of the cornea plays a critical role in maintaining this optical clarity. In the presence of infections, inflammation, trauma, burns, or other insults, blood or lymphatic vessels may invade the cornea, thereby leading to vision loss. LUT is recognized as a potent antioxidant, anti-inflammatory, and antiangiogenic agent following alkali injury in experimental animal models [39]. However, despite its promising pharmacological activities, the therapeutic potential of LUT is often limited by its poor water solubility and low bioavailability. To improve the bioavailability of LUT and achieve better therapeutic outcomes, we developed a novel LUT-loaded nanostructured lipid carrier-based in situ gel (LUT-NLC-ISG) and evaluated its in vitro and in vivo performance characteristics.

To increase the drug loading content of LUT in the formulation, a lipid phase comprising Mon and Cap was adopted, while RH 40 combined with PEG 400 was selected as the surfactant and cosurfactant system to optimize both solubility and stability parameters. These lipids are already approved by European and US regulatory authorities for topical application, are generally recognized as safe pharmaceutical adjuvants [40], and have well-established use in various dosage forms.

NLC were initially investigated to overcome the limitations of SLN, which have poor drug-loading capacity owing to their perfectly arranged crystalline structure, leading to drug expulsion during storage due to lipid crystallization [41]. The addition of liquid lipid (which is liquid at room temperature) to the formulation offers two main benefits: first, it creates a less ordered crystalline structure that provides additional space for drug loading; second, it reduces the crystallinity of the matrix and prevents drug expulsion. Typically, the proportion of liquid lipid in NLC formulations can be as high as 30% of the total lipid content [42,43,44]. In our developed formulation, the ratio of solid to liquid lipid is 4:6, which remains solid at 38 °C.

A larger microemulsion region in a pseudoternary phase diagram indicates stronger microemulsion-forming capacity of the formulation [45]. Pseudoternary phase diagrams can be used to determine the ratio of surfactant to cosurfactant; furthermore, the application of CCD-RSM can simultaneously reveal the interactions among different variables affecting the response values [46]. Using this approach, an optimized LUT-NLC-ISG formulation was developed, exhibiting a small droplet size of 25.27 ± 0.23 nm and a PDI of 0.24 ± 0.04. The optimized formulation remained stable at room temperature (25 °C) and under accelerated storage conditions for four weeks, showing acceptable changes in pH, clarity, droplet size, PDI, zeta potential, and drug loading content.

The in situ gelation mechanism involves a stimuli-responsive phase transition, in which the instilled solution transforms into a gel within the ocular cul-de-sac upon exposure to specific environmental triggers [33]. This drug delivery system combines the ease of administration inherent to liquid formulations with the therapeutic advantages of gel-mediated prolonged ocular retention, thereby improving upon conventional ophthalmic solutions by enhancing precorneal residence time and bioavailability. The sol–gel transition can be triggered by pH or the ionic strength of tear fluid. Notably, ionic strength-activated systems demonstrate superior efficacy among in situ gel-forming mechanisms, as they eliminate potential complications associated with pH and temperature fluctuations that can affect gelation consistency. GG is an ion-activated polymer that ensures consistent gelation performance and enhanced patient comfort [21]. Furthermore, CB exhibits enhanced mucoadhesive properties compared to GG [17], and the synergistic combination of GG and CB significantly improves the gelation characteristics of the in situ gel system. Using CCD again, we successfully developed an innovative in situ gel-forming ophthalmic solution containing NLC and incorporating GG and CB as dual pH- and ion-activated polymers. The addition of GG and CB to the formulation not only prolongs precorneal residence time but also hinders NLC aggregation. It has been previously reported that the high viscosity of polymers can increase the long-term physical stability of nanoparticles, which may be attributed to the stabilizing effect of the hydrogel network during storage [47].

The bio-adhesive force of the ISG formulation was determined to be 2231.7 ± 176.8 dyne/cm^2^ dyne/cm^2^, which was significantly higher than the shear force of the tear film (150 dyne/cm^2^; *p* < 0.05) [21]. The high bio-adhesive force of ISG increases corneal contact time and prevents the formulation from being readily washed away by the eye’s protective mechanisms [48]. The rheological properties of LUT-NLC-ISG enable a three-phase ocular drug delivery optimization: (1) liquid-state administration via standard droppers, (2) immediate gel formation with mucoadhesive retention (5094 mPa·s at rest), and (3) shear-responsive viscosity reduction during blinking (0.03 s^−1^) to minimize mechanical irritation while maintaining therapeutic residence time [49]. These viscoelastic properties—particularly the temperature- and stress-dual responsive behavior—confirm the ability of ISG to overcome the limitations of traditional eye drops by enabling spatiotemporal control of drug release kinetics and ocular surface interactions, ultimately enhancing bioavailability through prolonged precorneal retention [17,50].

The in vitro drug release profiles of LUT-Susp, LUT-NLC-ISG, and LUT-NLC in STF revealed that approximately 50% of LUT was cumulatively released from all three formulations by 72 h. However, between 24 h and 72 h, less than 10% of LUT was released from the LUT-Susp group, highlighting its characteristic rapid initial release followed by insufficient sustained release. In contrast, the LUT-NLC-ISG group demonstrated improved sustained release behavior, with approximately 20% of LUT released during the same 24–72 h period. An analysis of the release mechanisms for LUT-Susp, LUT-NLC, and LUT-NLC-ISG indicated that, among four kinetic models, the first-order equation best fit the release patterns of all three formulations.

Corneal permeability, as a key rate-limiting step in the bioavailability of topically applied ophthalmic formulations, is particularly important for poorly soluble drugs. The isolated corneal permeation assay showed that drug penetration in the LUT-NLC-ISG group was superior to that in the LUT-NLC and LUT-Susp groups after 15 min of administration, and a significant advantage was observed after 1 h (*p* < 0.05), demonstrating excellent corneal penetration performance. Ocular pharmacokinetic studies demonstrated that LUT-NLC-ISG improved drug bioavailability in the cornea, conjunctiva, and tear fluid compared with LUT-NLC and LUT-Susp. The pharmacokinetic characteristics of LUT-NLC-ISG are presented in Figure 4A. In addition to the fact that the inclusion of CB and GG in the NLC formulation induces in situ gel formation upon application to the conjunctival sac—thereby promoting LUT retention in the precorneal area—another contributing factor is the ability of CB to cross-link with mucins in the tear film mucus layer, which enhances corneal adhesion and prolongs the residence time of the ISG. Moreover, the small particle size of the NLC (less than 40 nm) enables faster corneal penetration [51]. Collectively, these characteristics contribute to improved therapeutic efficacy against CNV. Notably, compared with previously published research involving intraperitoneal injection of 200 mg/kg LUT, topical ocular administration of LUT-NLC and LUT-NLC-ISG achieved significantly higher drug levels in the corneal and conjunctival tissues than intraperitoneal injection (*p* < 0.01) [9].

In this study, a mouse model of corneal neovascularization (CNV) was established by alkali burn injury and used to evaluate the antiangiogenic effect of LUT-NLC-ISG. The antiangiogenic effects of LUT-NLC-ISG were confirmed by assessing the area of neovascularization. Furthermore, based on the analysis of hematoxylin and eosin-stained sections, LUT-NLC-ISG treatment reduced the number of new blood vessels. At the molecular level, topical ocular application of 0.1% LUT-NLC-ISG inhibited the protein expression of VEGF-A and MMP-9. Vascular endothelial growth factor receptors have been confirmed to be the predominant mediators of VEGF-stimulated endothelial cell migration, proliferation, and increased vascular permeability [52]. It is well established that LUT inhibits VEGF-A-mediated angiogenesis through multiple pathways. MMP-9 plays a critical role in corneal stromal degradation, and its expression is markedly upregulated after alkali burn injury [9]. High expression of MMP-9 is also closely associated with increased microvessel density, suggesting that it promotes angiogenesis in conjunction with VEGF-A [53]. Moreover, the increase in MMP-9 contributes to structural destabilization and functional impairment of corneal tissue through excessive proteolysis of the extracellular matrix. The group treated with 0.1% LUT-NLC-ISG (Group H) showed no significant difference from the DEX group, suggesting that LUT is a potential alternative to DEX as an anti-CNV agent.

## 5. Conclusions

This study developed a novel ophthalmic delivery system, LUT-NLC-ISG, for the treatment of corneal neovascularization (CNV). By integrating nanostructured lipid carriers (NLC) with a dual-responsive polymer matrix (GG and CB), the formulation achieved pH- and ion-triggered gelation, thereby prolonging precorneal residence time. Ex vivo corneal permeation and in vivo pharmacokinetic analyses revealed significantly higher drug flux and ocular bioavailability compared with LUT-NLC and LUT-Susp. Safety evaluations confirmed excellent biocompatibility with no observable ocular irritation under the tested conditions. The LUT-NLC-ISG (0.1%) suppressed neovascularization in a murine model of alkali burn-induced CNV, showing efficacy comparable to dexamethasone (0.025%) at the tested dose, an effect associated with downregulation of VEGF-A and MMP-9 expression. These findings suggest that LUT-NLC-ISG is a promising non-invasive candidate for CNV management, with the potential to address key challenges related to drug solubility, ocular retention, and therapeutic efficacy. Future studies should focus on long-term stability, clinical translation, and exploring more ophthalmic applications.

## Figures and Tables

**Table 1:** Composition and observed responses in CCD-RSM for LUT-NLC (X_1_: weight of mixed lipids; X_2_: the total weight of surfactant and cosurfactant; Y_1_: drug loading; Y_2_: droplet size).

Formulation	X_1_ (g)	X_2_ (g)	Y_1_ (%)	Y_2_ (nm)
1	0.259	1.459	0.96	13.55
2	0.541	1.459	0.80	14.17
3	0.259	1.741	0.98	15.21
4	0.541	1.741	0.86	17.67
5	0.2	1.6	1.10	14.17
6	0.6	1.6	0.76	15.12
7	0.4	1.4	0.91	17.98
8	0.4	1.8	1.03	15.58
9	0.4	1.6	0.94	14.975
10	0.4	1.6	0.94	14.95
11	0.4	1.6	0.95	14.57
12	0.4	1.6	0.95	14.43
13	0.4	1.6	0.95	14.56

**Table 2:** Composition and observed responses in CCD-RSM for in situ gel (X_3_: Concentration of CB (%, *w*/*v*); X_4_: Concentration of GG (%, *w*/*v*); V_1_: viscosity under the non-physiological condition (25 °C, pH 4–4.5, without STF); V_2_: viscosity at physiological condition (35 °C, with STF)).

Formulation	X_3_	X_4_	V_1_ (cP)	V_2_ (cP)	V_2_ − V_1_ (cP)
1	0.159	0.232	154.6	213.6	59
2	0.441	0.232	728.2	2223	1494.8
3	0.159	0.868	6796	5282	−1514
4	0.441	0.868	13,100	13,130	30
5	0.1	0.55	11,200	3806	−7394
6	0.5	0.55	7845	5660	−2185
7	0.3	0.1	233	213.6	−19.4
8	0.3	1	13,120	7884	−5236
9	0.3	0.55	6437	4010	−2427
10	0.3	0.55	6398	4379	−2019
11	0.3	0.55	6534	4019	−2515
12	0.3	0.55	6544	3874	−2670
13	0.3	0.55	6602	4097	−2505

**Table 3:** The mathematical models are fitting for LUT-NLC-ISG, LUT-NLC- and LUT-Susp.

	LUT-NLC-ISG		LUT-NLC		LUT-Susp	
	Equation	R^2^	Equation	R^2^	Equation	R^2^
Zero-order	Q = 4.288 + 0.701x	0.948	Q = 6.811 + 0.740t	0.908	Q = 9.362 + 0.863t	0.807
First Order	Q = 57.302 * ×(1 − e(−0.026x))	0.998	Q = 55.469 × (1 − e(−0.036x))	0.993	Q = 60.649 ×(1 − e(−0.050x))	0.991
Higuchi	Q = 6.473 × (x^1/2^) − 5.754	0.990	Q = 6.948 × (x^1/2^)−4.205	0.983	Q = 8.372 × (x^1/2^) − 4.466	0.942
Korsmeyer-Peppas	Q = 2.900 × (x^0.671^)	0.989	Q = 4.472 × (x^0.589^)	0.977	Q = 0.029×(x^0.540^)	0.930

**Table 4:** Ocular pharmacokinetics parameters after one single dose of LUT-NLC-ISG, LUT-NLC and LUT-Susp topical application (n = 6, mean ± SD).

Tissue	Pharmacokinetic Parameters	Unit	LUT-NLC-ISG	LUT-NLC	LUT-Susp
Conjunctiva	C_max_	μg/g	2.88 ± 1.66	0.31 ± 0.04	1.26 ± 0.36
	AUC_(0-t)_	μg/g*min	77.43	30.12	24.07
	MRT_(0-t)_	min	35.40	86.56	22.77
Cornea	C_max_	μg/g	1.06 ± 0.30	0.35 ± 0.04	0.18 ± 0.10
	AUC_(0-t)_	μg/g·min	104.15	47.92	24.36
	MRT_(0-t)_	min	69.41	86.31	78.97
Tears	C_max_	μg/g	178.06 ± 94.74	8.13 ± 1.35	201.29 ± 79.74
	AUC_(0-t)_	μg/g·min	3178.57	300.01	5802.81
	MRT_(0-t)_	min	15.12	71.10	38.83

## Data Availability

The data presented in this study are available on request from the corresponding author. The data are not publicly available as it was originally produced through research.

## Supplementary Materials

The following supporting information can be downloaded at: https://www.mdpi.com/article/10.3390/pharmaceutics18080908/s1. Figure S1 Specificity assessment of LUT in Cornea, Conjunctiva and Tear fluid. Figure S2 Standard curves of LUT in different tissues: (A) Tear fluid, (B) Conjunctiva, (C) Cornea. Table S1 Intra-batch assay and Inter-batch assay precision and accuracy for LUT. Table S2 Stability of LUT in different tissue samples. Table S3 Extraction recoveries of LUT from different tissues (mean ± SD, n=5). Figure S3 Rheological evaluation of LUT-NLC-ISG and LUT-NLC-ISG+STF: (A) Viscosity versus shear rate; (B) Shear stress versus shear rate. Table S4 Viscosity of LUT-NLC-ISG versus shear rate. Table S5 Shear stress versus shear rate. Figure S4 Kinetic models fitted to the drug release data: (A) Zero order, (B) First order, (C) Higuchi, (D) Korsmeyer-Peppas. Figure S5 Histogram of particle size distribution of (A) blank-NLC; (B) LUT-NLC [54,55,56].

## Abbreviations

The following abbreviations are used in this manuscript:

AUC	area under the concentration–time curve
Cap	Capryol 90
CB	Carbopol ETD 2020
CCK-8	Cell Counting Kit-8
CCL-20.2	conjunctival epithelial cell line CCL-20.2
CCD-RSM	central composite design-response surface methodology
CNV	corneal neovascularization
DC	drug concentration
DEX	dexamethasone
DL	drug loading
DS	droplet size
DSC	differential scanning calorimetry
EE	encapsulation efficiency
ELISA	enzyme-linked immunosorbent assay
FBS	fetal bovine serum
GG	gellan gum
H&E	hematoxylin and eosin
HCE-2	human corneal epithelial cell line
CCL-20.2	human conjunctival epithelial cells
HET-CAM	hen’s egg test–chorioallantoic membrane assay
HL	hydration level
ISG	in situ gel
Jss	steady-state flux
LUT	luteolin
MMP-9	matrix metalloproteinase-9
Mon	monostearin
NLC	nanostructured lipid carriers
Papp	apparent permeability coefficient
PDI	polydispersity index
PM	physical mixture
RH40	PEG-40 hydrogenated castor oil
SEM	scanning electron microscopy
SLN	solid lipid nanoparticles
STF	simulated tear fluid
TEM	transmission electron microscopy
VEGF-A	vascular endothelial growth factor A
ZP	zeta potential

## References

[1] Sun M., Wang J., Fan W., Wang K., Wang Y., Zhang J., Ding S., Zhang C., Feng Z., Wang Y.-G. (2025). From Design to Deployment: The Innovative Role of Nanomicelles in Targeted Ophthalmic Therapeutics. Mater. Today Bio.

[2] Salica J.P., Potilinski M.C., Ortiz G., Maffia P.C., Guerrieri D., Chuluyan E., Gallo J.E. (2026). Downregulation of the Transglutaminase 2-NF-κB Inflammatory Axis by a Fusion Protein of Cementoin and Secretory Leukocyte Protease Inhibitor Reduces Corneal Angiogenesis. Int. J. Mol. Sci..

[3] Liu X., Wang S., Wang X., Liang J., Zhang Y. (2017). Recent Drug Therapies for Corneal Neovascularization. Chem. Biol. Drug Des..

[4] Chan P.S., Li Q., Zhang B., To K.K.W., Leung S.S.Y. (2020). In Vivo Biocompatibility and Efficacy of Dexamethasone-Loaded PLGA-PEG-PLGA Thermogel in an Alkali-Burn Induced Corneal Neovascularization Disease Model. Eur. J. Pharm. Biopharm..

[5] Chung A.S., Ferrara N. (2011). Developmental and Pathological Angiogenesis. Annu. Rev. Cell Dev. Biol..

[6] Zhu H., Ye J., Wu Y., Cheng Y., Su M., Dai Q., Han Y., Pan J., Wu Z., Chen C. (2024). A Synergistic Therapy with Antioxidant and Anti-VEGF: Toward Its Safe and Effective Elimination for Corneal Neovascularization. Adv. Healthc. Mater..

[7] Joussen A.M., Rohrschneider K., Reichling J., Kirchhof B., Kruse F.E. (2000). Treatment of Corneal Neovascularization with Dietary Isoflavonoids and Flavonoids. Exp. Eye Res..

[8] Omran S., Elnaggar Y.S.R., Abdallah O.Y. (2024). Controlled Release, Chitosan-Tethered Luteolin Phytocubosomes; Formulation Optimization to in-Vivo Antiglaucoma and Anti-Inflammatory Ocular Evaluation. Int. J. Biol. Macromol..

[9] Wang H., Guo Z., Liu P., Yang X., Li Y., Lin Y., Zhao X., Liu Y. (2023). Luteolin Ameliorates Cornea Stromal Collagen Degradation and Inflammatory Damage in Rats with Corneal Alkali Burn. Exp. Eye Res..

[10] Park S.W., Cho C.S., Jun H.O., Ryu N.H., Kim J.H., Yu Y.S., Kim J.S., Kim J.H. (2012). Anti-Angiogenic Effect of Luteolin on Retinal Neovascularization via Blockade of Reactive Oxygen Species Production. Invest. Ophthalmol. Vis. Sci..

[11] Shakeel F., Alamer M.M., Alam P., Alshetaili A., Haq N., Alanazi F.K., Alshehri S., Ghoneim M.M., Alsarra I.A. (2021). Hepatoprotective Effects of Bioflavonoid Luteolin Using Self-Nanoemulsifying Drug Delivery System. Molecules.

[12] Irimia T., Ghica M.V., Popa L., Anuţa V., Arsene A.-L., Dinu-Pîrvu C.-E. (2018). Strategies for Improving Ocular Drug Bioavailability and Corneal Wound Healing with Chitosan-Based Delivery Systems. Polymers.

[13] Krstić L., González-García M.J., Diebold Y. (2021). Ocular Delivery of Polyphenols: Meeting the Unmet Needs. Molecules.

[14] Beloqui A., Solinís M.Á., Rodríguez-Gascón A., Almeida A.J., Préat V. (2016). Nanostructured Lipid Carriers: Promising Drug Delivery Systems for Future Clinics. Nanomedicine.

[15] Xie M., Wang H., Gao T., Peng J., Meng P., Zhang X., Guo D., Liu G., Shi J., Peng Q. (2023). The Protective Effect of Luteolin on the Depression-Related Dry Eye Disorder through Sirt1/NF-κB/NLRP3 Pathway. Aging.

[16] Lin H.R., Sung K.C. (2000). Carbopol/Pluronic Phase Change Solutions for Ophthalmic Drug Delivery. J. Control Release.

[17] Ranch K.M., Maulvi F.A., Naik M.J., Koli A.R., Parikh R.K., Shah D.O. (2019). Optimization of a Novel in Situ Gel for Sustained Ocular Drug Delivery Using Box-Behnken Design: In Vitro, Ex Vivo, in Vivo and Human Studies. Int. J. Pharm..

[18] Gonzalez-Mira E., Nikolić S., Calpena A.C., Egea M.A., Souto E.B., García M.L. (2012). Improved and Safe Transcorneal Delivery of Flurbiprofen by NLC and NLC-Based Hydrogels. J. Pharm. Sci..

[19] Coneac G., Vlaia V., Olariu I., Muţ A.M., Anghel D.F., Ilie C., Popoiu C., Lupuleasa D., Vlaia L. (2015). Development and Evaluation of New Microemulsion-Based Hydrogel Formulations for Topical Delivery of Fluconazole. AAPS PharmSciTech.

[20] Lee D., Gutowski I.A., Bailey A.E., Rubatat L., de Bruyn J.R., Frisken B.J. (2011). Investigating the Microstructure of a Yield-Stress Fluid by Light Scattering. Phys. Rev. E Stat. Nonlin Soft Matter Phys..

[21] Kotreka U.K., Davis V.L., Adeyeye M.C. (2017). Development of Topical Ophthalmic In Situ Gel-Forming Estradiol Delivery System Intended for the Prevention of Age-Related Cataracts. PLoS ONE.

[22] Gugleva V., Mihaylova R., Kamenova K., Zheleva-Dimitrova D., Stefanova D., Tzankova V., Zaharieva M.M., Najdenski H., Forys A., Trzebicka B. (2024). Development and Characterization of Dual-Loaded Niosomal Ion-Sensitive In Situ Gel for Ocular Delivery. Gels.

[23] Demarinis G., Tatti F., Taloni A., Giugliano A.V., Panthagani J., Myerscough J., Peiretti E., Giannaccare G. (2023). Treatments for Ocular Diseases in Pregnancy and Breastfeeding: A Narrative Review. Pharmaceuticals.

[24] Adısanoğlu P., Özgüney I. (2024). Development and Characterization of Thermosensitive and Bioadhesive Ophthalmic Formulations Containing Flurbiprofen Solid Dispersions. Gels.

[25] Eldesouky L.M., El-Moslemany R.M., Ramadan A.A., Morsi M.H., Khalafallah N.M. (2021). Cyclosporine Lipid Nanocapsules as Thermoresponsive Gel for Dry Eye Management: Promising Corneal Mucoadhesion, Biodistribution and Preclinical Efficacy in Rabbits. Pharmaceutics.

[26] Shastri D.H., Prajapati S.T., Patel L.D. (2010). Design and Development of Thermoreversible Ophthalmic In Situ Hydrogel of Moxifloxacin HCl. Curr. Drug Deliv..

[27] Nirbhavane P., Sharma G., Singh B., Begum G., Jones M.-C., Rauz S., Vincent R., Denniston A.K., Hill L.J., Katare O.P. (2020). Triamcinolone Acetonide Loaded-Cationic Nano-Lipoidal Formulation for Uveitis: Evidences of Improved Biopharmaceutical Performance and Anti-Inflammatory Activity. Colloids Surf. B Biointerfaces.

[28] Bagul U.S., Khot S.V., Ashtekar K.S., Monde A.A., Kolhe O.H., Tagalpallewar A.A., Kokare C.R. (2024). Fabrication of Acetazolamide Loaded Leciplex for Intraocular Delivery: Optimization by 32 Full Factorial Design, in Vitro, Ex Vivo and in Vivo Pharmacodynamics. Int. J. Pharm..

[29] Yu L., Meng Q., Li M., Tian L., Wu X., Jie Y. (2025). Heating-Driven Self-Assembled Glycyrrhizin Nanomicelles Loading Bisdemethoxycurcumin: Preparation, Characterization, and Efficacy Evaluation on Experimental Dry Eye. Colloids Surf. B Biointerfaces.

[30] Irani Y.D., Scotney P.D., Klebe S., Mortimer L.A., Nash A.D., Williams K.A. (2017). An Anti-VEGF-B Antibody Fragment Induces Regression of Pre-Existing Blood Vessels in the Rat Cornea. Investig. Ophthalmol. Vis. Sci..

[31] Elmanawy M.A., Boraie N., Bakr B.A., Makled S. (2024). Augmented Ocular Uptake and Anti-Inflammatory Efficacy of Decorated Genistein-Loaded NLCs Incorporated in in Situ Gel. Int. J. Pharm..

[32] Moustafa M.A., El-Refaie W.M., Elnaggar Y.S.R., Abdallah O.Y. (2018). Gel in Core Carbosomes as Novel Ophthalmic Vehicles with Enhanced Corneal Permeation and Residence. Int. J. Pharm..

[33] Adwan S., Obeidi T., Al-Akayleh F. (2024). Chitosan Nanoparticles Embedded in In Situ Gel for Nasal Delivery of Imipramine Hydrochloride: Short-Term Stage Development and Controlled Release Evaluation. Polymers.

[34] Abdessemed M., Bouacida S., Turki M., Ben Haj Koubaier H., Omrani S., Allouache R., Bouzouita N., Karoui R., Snoussi A. (2024). Chemical Characterization and Biological Activities Evaluation of Myrtus Communis L. Essential Oil Extraction By-Product towards Circular Economy and Sustainability. Foods.

[35] Wang M., Yang Y., Li D., Wang Y., Ji T., Li Q., Zhang J., Zhang P., Su J. (2025). Miconazole-Splitomicin Combined β-Glucan Hydrogel for Effective Prevention of Candida Albicans Periprosthetic Joint Infection. Eur. J. Pharm. Sci..

[36] Schroeder S.L.M. (2026). Eyring-Polanyi Rate Theory for the Homogeneous Nucleation of Organic Crystals from Solution. Cryst. Growth Des..

[37] Civir G.O., Bahadori F., Ozay O., Ergin Kızılçay G., Atesoglu S., Aldogan E.H., Celik B. (2025). Design and In Vitro Evaluation of Cross-Linked Poly(HEMA)-Pectin Nano-Composites for Targeted Delivery of Potassium Channel Blockers in Cancer Therapy. Gels.

[38] Luo Q., Yang J., Xu H., Shi J., Liang Z., Zhang R., Lu P., Pu G., Zhao N., Zhang J. (2022). Sorafenib-Loaded Nanostructured Lipid Carriers for Topical Ocular Therapy of Corneal Neovascularization: Development, in-Vitro and in Vivo Study. Drug Deliv..

[39] Chen Y.-F., Wu S., Li X., Chen M., Liao H.-F. (2022). Luteolin Suppresses Three Angiogenesis Modes and Cell Interaction in Uveal Melanoma in Vitro. Curr. Eye Res..

[40] Sánchez-López E., Espina M., Doktorovova S., Souto E.B., García M.L. (2017). Lipid Nanoparticles (SLN, NLC): Overcoming the Anatomical and Physiological Barriers of the Eye—Part I—Barriers and Determining Factors in Ocular Delivery. Eur. J. Pharm. Biopharm..

[41] Gugleva V., Andonova V. (2023). Recent Progress of Solid Lipid Nanoparticles and Nanostructured Lipid Carriers as Ocular Drug Delivery Platforms. Pharmaceuticals.

[42] Scioli Montoto S., Muraca G., Ruiz M.E. (2020). Solid Lipid Nanoparticles for Drug Delivery: Pharmacological and Biopharmaceutical Aspects. Front. Mol. Biosci..

[43] Elmowafy M., Al-Sanea M.M. (2021). Nanostructured Lipid Carriers (NLCs) as Drug Delivery Platform: Advances in Formulation and Delivery Strategies. Saudi Pharm. J..

[44] Shahzadi I., Fürst A., Knoll P., Bernkop-Schnürch A. (2021). Nanostructured Lipid Carriers (NLCs) for Oral Peptide Drug Delivery: About the Impact of Surface Decoration. Pharmaceutics.

[45] Mehanna M.M., Abla K.K., Elmaradny H.A. (2021). Tailored Limonene-Based Nanosized Microemulsion: Formulation, Physicochemical Characterization and In Vivo Skin Irritation Assessment. Adv. Pharm. Bull..

[46] Wang X., Liu J., Ma Y., Cui X., Chen C., Zhu G., Sun Y., Tong L. (2023). Development of A Nanostructured Lipid Carrier-Based Drug Delivery Strategy for Apigenin: Experimental Design Based on CCD-RSM and Evaluation against NSCLC In Vitro. Molecules.

[47] Chen H., Xiao L., Du D., Mou D., Xu H., Yang X. (2009). A Facile Construction Strategy of Stable Lipid Nanoparticles for Drug Delivery Using a Hydrogel-Thickened Microemulsion System. Nanotechnology.

[48] Alsaidan O.A., Zafar A., Yasir M., Alzarea S.I., Alqinyah M., Khalid M. (2022). Development of Ciprofloxacin-Loaded Bilosomes In-Situ Gel for Ocular Delivery: Optimization, In-Vitro Characterization, Ex-Vivo Permeation, and Antimicrobial Study. Gels.

[49] Lee C.H., Moturi V., Lee Y. (2009). Thixotropic Property in Pharmaceutical Formulations. J. Control Release.

[50] Ludwig A., van Haeringen N.J., Bodelier V.M., Van Ooteghem M. (1992). Relationship between Precorneal Retention of Viscous Eye Drops and Tear Fluid Composition. Int. Ophthalmol..

[51] Niamprem P., Srinivas S.P., Tiyaboonchai W. (2019). Penetration of Nile Red-Loaded Nanostructured Lipid Carriers (NLCs) across the Porcine Cornea. Colloids Surf. B Biointerfaces.

[52] Nicholas M.P., Mysore N. (2021). Corneal Neovascularization. Exp. Eye Res..

[53] Ochirbat S., Kan T.-C., Hsu C.-C., Huang T.-H., Chuang K.-H., Chen M., Cheng C.-C., Chang C.-C., Rahayu S., Chang J. (2024). The Angiogenic Role of the Alpha 9-Nicotinic Acetylcholine Receptor in Triple-Negative Breast Cancers. Angiogenesis.

[54] Shi J., Yang J., Xu H., Luo Q., Sun J., Zhang Y., Liang Z., Zhao N., Zhang J. (2023). Preparation of a Sunitinib Loaded Microemulsion for Ocular Delivery and Evaluation for the Treatment of Corneal Neovascularization in Vitro and in Vivo. Front. Pharmacol..

[55] Peira E., Chindamo G., Chirio D., Sapino S., Oliaro-Bosso S., Rebba E., Ivanchenko P., Gallarate M. (2021). Assessment of In-Situ Gelling Microemulsion Systems upon Temperature and Dilution Condition for Corneal Delivery of Bevacizumab. Pharmaceutics.

[56] Destruel P.-L., Zeng N., Maury M., Mignet N., Boudy V. (2017). In Vitro and in Vivo Evaluation of in Situ Gelling Systems for Sustained Topical Ophthalmic Delivery: State of the Art and Beyond. Drug Discov. Today.

