# Supplementary material for: Ion- and pH-Responsive In Situ Gel Incorporating Luteolin-Loaded Nanostructured Lipid Carriers Enhances Ocular Bioavailability and Anti-Angiogenic Efficacy for Corneal Neovascularization
Source: Pharmaceutics. 2026 Jul 24;18(8):908. doi: 10.3390/pharmaceutics18080908 (PMC13517062; doi:10.3390/pharmaceutics18080908)
Supplement: Supplementary file 1 [file pharmaceutics-18-00908-s001.zip › pharmaceutics-4359271-supplementary.pdf]

pharmaceutics

Article

Supplementary Materials: Ion- and pH-responsive *in situ* gel incorporating luteolin-loaded nanostructured lipid carriers enhances ocular bioavailability and anti-angiogenic efficacy for corneal neovascularization

Section S1 Method validation parameters for the bioanalytical methods

1. Method validation parameters for the bioanalytical methods

1.1 Preparation of Stock and Standard Solutions

An analytical balance was used to weigh 9.30 mg of LUT into a 10 mL volumetric flask, and the volume was made up with methanol to obtain a 0.930 mg/mL LUT stock solution. Using a pipette and volumetric flasks of various capacities, the stock solution was serially diluted with methanol to prepare standard solutions (working standard concentration ranges for tear fluid, cornea, and conjunctiva: 1860, 930, 310, 155, 51.67, and 25.83 ng/mL). Aliquots of 100 μL of each LUT standard solution were transferred into glass centrifuge tubes and evaporated to dryness under a stream of nitrogen at room temperature, then set aside for later use [1].

1.2 Specificity

Blank matrix preparation: Exactly 400 μL of methanol was measured and used to extract corneal tissue. After sealing, the sample was maintained under constant-temperature conditions (4 ± 2 °C) for 24 h to obtain the corneal extract.

Standard preparation: The 100 ng/mL dried standard residues was mixed with the corneal blank matrix to prepare a 51.67 ng/mL LUT cornea-specific standard.

Test sample groups: Included blank corneal extract, 51.67 ng/mL LUT standard, blank corneal extract spiked with LUT-NLC ISG composite solution (51.67 ng/mL), and extracts of the corneal sample after administration of 0.1% LUT NLC ISG. All samples were centrifuged at high speed (12,000 rpm, 10 min), and the supernatants were injected for HPLC analysis to specifically verify whether endogenous components in the corneal matrix interfere with the detection of the target analyte. The processing of conjunctival samples and tear fluid sample were the same as that of corneas.

As shown in Supplementary Figure S1, the retention time of LUT in all tissue matrices was 4.20 minutes. In each tissue sample, the impurity peaks did not elute simultaneously with the LUT peaks, indicating that the endogenous components in each tissue did not interfere with the quantitative analysis of LUT. These results indicate that the developed method has good specificity.

 https://doi.org/10.3390/pharmaceutics18080908

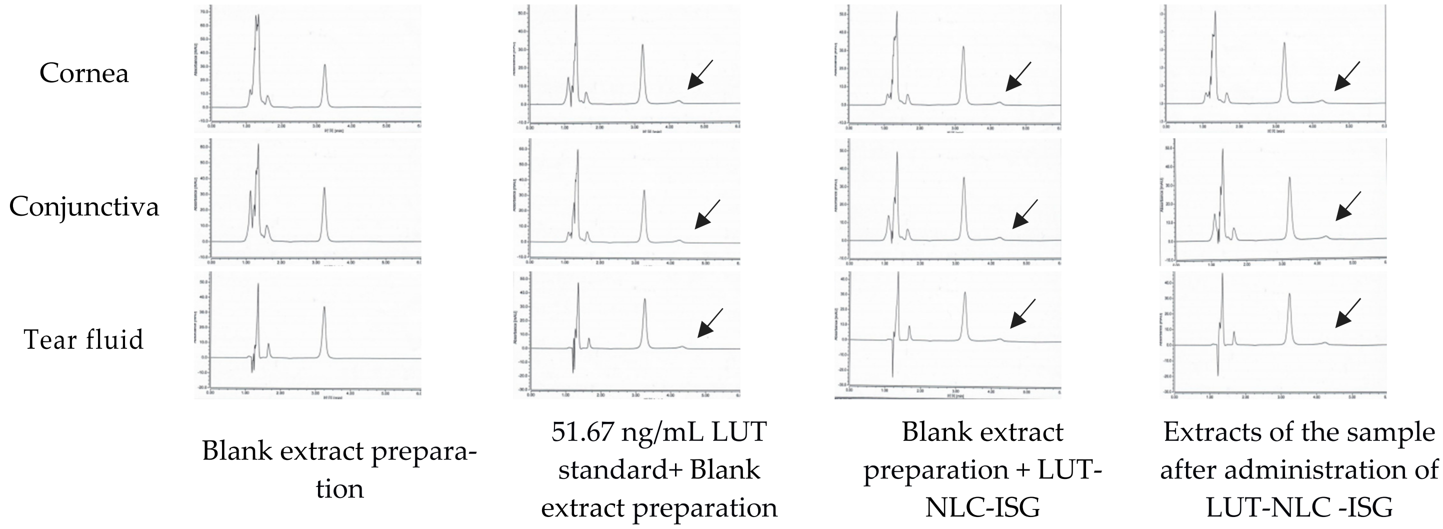

Supplementary Figure S1 Specificity assessment of LUT i n Cornea, Conjunctiva and Tear fluid.

1.3 Linearity

The dried standard residues (1860, 930, 310, 155, 51.67, and 25.83 ng/mL) were reconstituted with 100 μL of corneal blank extract. After vortex mixing, the samples were centrifuged at 12,000 rpm for 10 min, and the supernatant was injected for HPLC analysis. The characteristic peak areas were recorded to construct a concentration-response curve (x-axis: concentration; y-axis: peak area). Linear regression was performed using the least-squares method, and the coefficient of determination (R^2) was calculated. Analysis of variance (ANOVA) was applied to verify the significance of regression and the deviation from linearity ($\alpha = 0.05$). The lower limit of quantification (LLOQ) was defined as the lowest concentration on the calibration curve that could be determined with accuracy and precision within ±20%.

The processing of conjunctival samples and tear fluid sample were the same as that of corneas.

Linear regression analysis yielded the following calibration curve equations:

Cornea: $Y = 0.0015X - 0.0198$ ($R^2 = 0.999$)

Conjunctiva: $Y = 0.0012X - 0.0137$ ($R^2 = 0.999$)

Tear fluid: $Y = 0.0014X - 0.0241$ ($R^2 = 0.999$)

The calibration curves are presented in Supplementary Figure S2. The results demonstrate that LUT exhibits good linearity over the concentration range of 25–1860 ng/mL in cornea, conjunctiva, and tear fluid. The LLOQ was determined to be 25.83 ng/mL for all three matrices.

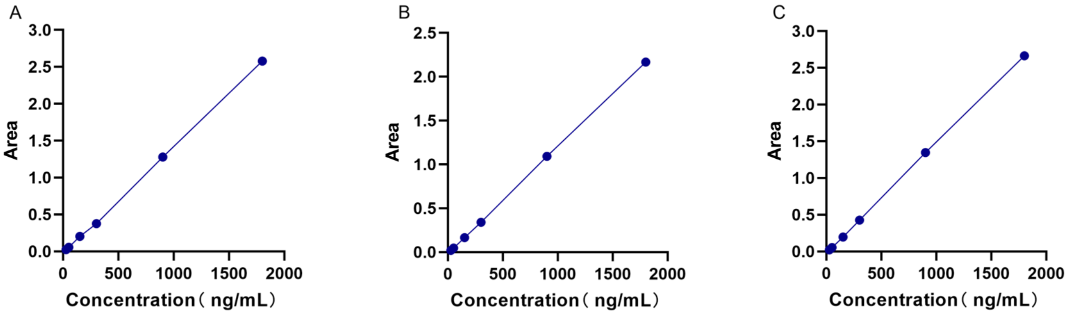

Supplementary Figure S2 Standard curves of LUT in different tissues: (A) Tear fluid, (B) Conjunctiva, (C) Cornea.

1.4 Precision and accuracy

Corneal samples, conjunctival samples, and tear fluid samples at four concentration levels (930, 310, 51.67, and 25.83 ng/mL) were prepared, with five replicates for each concentration. HPLC was used to perform intra-day precision testing (five replicate injections on the same day) and inter-day precision validation over three consecutive days. The data were processed to calculate recovery (Re = measured value/theoretical value × 100%) and relative standard deviation (RSD). The lower limit of quantification (LLOQ) was required to meet the acceptance criteria of signal-to-noise ratio (S/N ≥ 10) and RSD ≤ 15%.

The precision results for each tissue are summarized in Supplementary Table S1. The RSD values at all tested concentrations were less than 5%, meeting the required acceptance criteria.

Supplementary Table S1 Intra-batch assay and Inter-batch assay precision and accuracy for LUT.

Concentration of LUT (ng/mL)		Intra-batch assay (n=5)						Inter-batch assay	
		1st day		2nd day		3rd day		%RSD	% RE
		%RSD	%RE	%RSD	%RE	%RSD	%RE		
Tear fluid	25.83	3.05	103.57	1.86	105.76	2.43	106.67	2.77	105.33
	51.67	3.42	101.92	3.88	96.00	2.84	91.81	4.49	96.58
	310.00	2.34	97.44	0.56	98.57	2.48	99.54	2.18	98.52
	930.00	0.80	92.51	1.66	93.74	2.49	92.69	1.88	92.98
Cornea	25.83	2.74	108.60	2.77	108.20	2.55	107.47	2.72	108.09
	51.67	3.62	101.70	2.96	104.90	1.86	105.10	3.26	103.90
	310.00	1.51	99.96	3.73	102.62	3.82	104.27	3.67	102.28
	930.00	2.81	102.90	1.67	103.51	3.98	103.27	2.99	103.23
Conjunctiva	25.83	0.81	102.63	1.96	105.43	1.61	105.09	1.96	104.38
	51.67	1.54	103.49	4.27	108.80	4.37	107.31	4.24	106.53
	310.00	3.27	91.87	2.11	94.43	3.91	93.24	3.78	93.18
	930.00	1.33	90.89	2.12	94.76	3.59	92.58	2.70	92.08

1.5 Sample Stability

Samples of blank corneal extract spiked with LUT-NLC ISG were prepared in triplicate at three nominal concentrations (930, 310, and 51.67ng/mL). Their stability was examined under the following three conditions: (i) storage in a temperature-controlled autosampler at 10 °C ± 2 °C

for 7 h to assess low temperature storage stability; (ii) storage at 25 °C for 2 h to assess room-temperature stability; and (iii) three complete freeze-thaw cycles, each consisting of freezing at -70 °C for 12 h followed by thawing at 25 °C. After each treatment, samples were analyzed by HPLC, and the measured concentrations were derived from the calibration curve. Recoveries (Re) and relative standard deviations (RSD) were computed from triplicate measurements. The identical protocol was followed for conjunctival and tear fluid matrices.

The stability results for LUT are presented in Supplementary Table S2. For all tested samples, the RSD values were below 5%, and the recovery (Re) values were within the acceptable range of 80–115%. These findings indicate that luteolin remains stable in tear fluid, cornea, and conjunctiva under the evaluated storage conditions (autosampler stability, room temperature stability, and freeze-thaw stability).

Supplementary Table S2 Stability of LUT in different tissue samples

Sample	Autosampler stability		Room-temperature stability		Freeze-thaw stability	
	%RSD	%RE	%RSD	%RE	%RSD	%RE
Tear fluid	4.53	103.88	1.96	99.81	1.58	99.56
	3.43	96.21	1.18	95.75	2.35	93.99
	1.05	93.55	1.13	92.81	0.44	92.14
Cornea	2.12	104.33	2.78	103.63	3.15	102.66
	3.30	97.73	3.12	95.05	1.49	94.34
	2.55	99.13	2.23	100.02	0.97	100.00
Conjunctiva	1.76	88.48	1.21	86.48	1.80	84.12
	1.66	94.92	0.51	95.08	4.38	91.01
	1.57	85.78	1.27	86.01	1.07	85.04

1. 6 Extraction Recovery

Corneal samples, conjunctival samples, and tear fluid samples at three concentration levels (930, 310, and 51.67 ng/mL) were prepared, with five replicates for each concentration. HPLC was used to determine the chromatographic peak areas of the experimental group (simulated sample solution, A1) and the chromatographic response of the control group (original concentration standard solution, A2). The apparent recovery was calculated by the peak area ratio method (A1/A2 × 100%).

The extraction recoveries of LUT from tear fluid, cornea, and conjunctiva at low, medium, and high concentrations (930, 310, and 51.67 ng/mL) are summarized in Supplementary Table S3. The recoveries ranged between 85% and 115% for all biological matrices, meeting the methodological validation requirements. This confirms that the HPLC method used for determining drug concentrations in these tissues is reliable.

Supplementary Table S3 Extraction recoveries of LUT from different tissues (mean ± SD, n=5)

Sample	Extraction recoveries (%)		
	51.67 ng/mL	310 ng/mL	930 ng/mL
Tear fluid	102.97±0.87	96.7±1.12	109.39±1.19
Cornea	98.74±3.57	95.54±1.31	95.27±4.89

Conjunctiva	110.55±1.41	94.19±1.87	98.32±0.38

Section S2 Rheological evaluation before and after simulated tear fluid exposure

2.1 Rheology

Rheological analysis was performed using a rotary rheometer equipped with a cone-plate geometry (cone diameter: 35 mm, cone angle: 2°). The shear stress of the formulation was measured over a range of shear rates under two conditions: at 25 ± 0.1 °C without STF (simulated pre-administration environment), and at 34 ± 0.1 °C with STF (simulated ocular surface environment). A typical run consisted of increasing the shear rate from 0 to 100 s^{-1} at a controlled ramp speed, with a 35 s hold at each shear rate. All measurements were performed in triplicate. The flow behavior of the formulation was determined from the plots of shear stress versus shear rate and viscosity versus shear rate.

To be suitable for use as a conventional eye drop, the formulation must possess adequate fluidity prior to administration. Meanwhile, the gel state is closely associated with sustained drug release and requires desirable viscoelastic and viscosity properties [2]. Therefore, the rheological behavior of LUT-NLC-ISG before and after administration directly reflects the key principles underlying its *in situ* gelation mechanism. In this study, rheological parameters, including shear stress and viscosity as functions of shear rate, were measured under non-physiological conditions (without STF, at 25 ± 0.5 °C) and physiological conditions (with STF, at 34 ± 0.5 °C).

As shown in Supplementary Figure S3B and Supplementary Table S5, under simulated pre-administration environment, shear stress increased linearly with increasing shear rate (R^2 = 0.9821), indicating Newtonian fluid behavior [3]. The initially low viscosity suggests that the formulation exhibits excellent flowability and comfort prior to administration. Under simulated ocular surface environment, the shear stress at a shear rate of 100 s^{-1} was 0.022 times that at 0.1 s^{-1}. Moreover, as depicted in Supplementary Figure S3A and Supplementary Table S4, viscosity decreased with increasing shear rate, demonstrating typical shear-thinning behavior [4], which indicates that LUT-NLC-ISG behaves as a non-Newtonian fluid under physiological conditions. Such rheological properties are beneficial for sustained drug release, uniform distribution over the ocular surface upon blinking, and reduction of ocular irritation caused by excessively high viscosity [5,6].

Furthermore, as shown in Supplementary Figure S3A, at a shear rate of 0.1 s^{-1} (close to the blink-associated shear rate of 0.03 s^{-1} [7]), the viscosity reached 5094 mPa·s, indicating that the formulation becomes viscous upon eye closure (150 mPa·s), thereby enabling effective retention on the ocular surface and preventing rapid clearance. Collectively, these results suggest that LUT-NLC ISG undergoes a significant phase transition, which is of great importance for enhancing the ocular bioavailability of the drug.

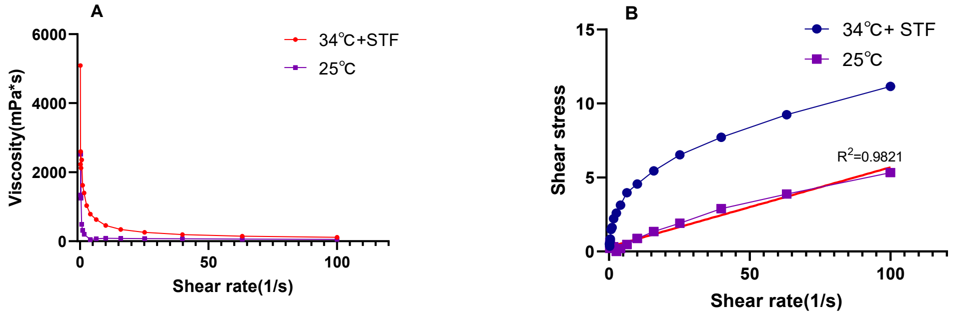

Supplementary Figure S3 Rheological evaluation of LUT-NLC-ISG and LUT-NLC-ISG+STF: (A) Viscosity versus shear rate; (B) Shear stress versus shear rate

Supplementary Table S4 Viscosity of LUT-NLC-ISG versus shear rate

Shear rate	Viscosity (34°C+STF, mPa*s)			Viscosity (25°C, mPa*s)		
(1/s)	1	2	3	1	2	3
0.099998	5093.83	5102.87	5094.34	-	-	-
0.158459	2229.17	2237.05	2235.83	1335.68	1338.26	1344.43
0.251268	2599.39	2605.96	2602.91	2521.15	2529.64	2525.75
0.398126	2124.97	2128.74	2132.39	1247.35	1252.36	1249.8
0.631046	2356.13	2357.61	2356.14	489.438	498.348	493.588
1.00012	1622.45	1626.43	1627.03	311.036	313.246	320.556
1.58511	1396.25	1405.26	1398.89	200.556	206.166	207.306
2.5122	1034.3	1036.82	1041.14	*150.674*	*164.357*	*157.648*
3.98138	785.021	789.251	789.271	108.174	112.476	108.9264
6.30986	629.494	630.124	631.994	94.3353	98.7153	90.7353
10.0006	455.83	458.23	459.47	87.9323	92.8823	97.0623
15.8499	343.422	348.772	343.482	84.7293	93.0693	91.0893
25.1204	259.976	263.446	265.536	76.0845	82.7245	84.6545
39.8128	193.941	201.241	194.371	72.6513	82.3213	76.2113
63.0995	146.509	153.919	152.999	61.5637	68.5337	66.3337
100.006	111.509	120.839	112.919	53.368	55.368	55.208

Supplementary Table S5 Shear stress versus shear rate

Shear rate	Shear stress (34°C+STF, Pa)			Shear stress (25°C, Pa)		
(1/s)	1	2	3	1	2	3
0.099998	0.509371	0.513401	0.509951	-	-	-
0.158459	0.353231	0.357801	0.354891	0.211479	0.217939	0.215699
0.251268	0.653144	0.658574	0.658444	0.633398	0.642488	0.638718
0.398126	0.846005	0.849345	0.849995	0.496672	0.499792	0.504722
0.631046	1.48683	1.49285	1.49543	0.308885	0.312495	0.314185
1.00012	1.62263	1.63166	1.62531	0.310958	0.311178	0.312018
1.58511	2.21322	2.21557	2.22238	0.31788	0.32103	0.32704
2.5122	2.59838	2.60638	2.60193	0.342267	0.014485	0.013465
3.98138	3.12547	3.13526	3.12846	0.409816	0.201303	0.192723

6.30986	3.97202	3.97777	3.98105	0.469022	0.476952	0.477502
10.0006	4.5586	4.56253	4.56609	0.879346	0.879846	0.881166
15.8499	5.44319	5.4481	5.44638	1.34284	1.351	1.34794
25.1204	6.53069	6.54019	6.53343	1.91112	1.92054	1.91324
39.8128	7.72132	7.72567	7.72966	2.89231	2.89838	2.89587
63.0995	9.24467	9.2517	9.25039	3.88437	3.8908	3.89105
100.006	11.1516	11.15265	11.16004	5.33677	5.34061	5.34622

Section S4 Kinetic models fitted to the drug release data

The in vitro release of LUT from LUT-NLC-ISG, LUT-NLC, and LUT-Susp (each containing 1 mg/mL) was evaluated using the dialysis bag method (MWCO 10 kDa) under sink conditions. Each formulation was placed in a dialysis bag and immersed in 100 mL of STF (pH 7.4, containing 0.25% Tween 80 to maintain sink conditions) at 37 °C with shaking at 100 rpm. At 0.5, 1, 2, 4, 6, 8, 12, 24, 36, 48, and 72h, 0.2 mL samples were withdrawn and replaced with an equal volume of fresh medium. The drug concentration was analyzed by HPLC in triplicate. Cumulative release percentages were calculated using the appropriate equation, and release kinetics were modeled using zero-order, first-order, Higuchi, and Korsmeyer-Peppas equations.

$$Q(\%) = \frac{C_n \mathrm{V} + \mathrm{V}_i \sum_{i=0}^{i=n} C_i}{\mathrm{W}_0} \times 100\%$$

Where W_0 is the initial amount of LUT loaded into the dialysis bag, C_n is the LUT concentration in the released medium at t_n, t_n is sampling at the Nth time, V is the total volume of the release medium (100 mL), V_i is the sample volume (0.2 mL) at t_i and C_i is the sample concentration at t_i.

As shown in Figure S4, the *in vitro* release of LUT from the LUT-NLC-ISG, LUT-NLC, and LUT-Susp best fits the first-order kinetic model with the highest correlation, indicating that the release process was concentration-dependent and predominantly governed by passive diffusion across the lipid matrix and the aqueous boundary layer [8]. This kinetic model implies that the rate of release is proportional to the concentration of LUT remaining in the formulation, which can be advantageous for maintaining therapeutic levels over an extended period [9]. Collectively, these findings confirm that the NLC-ISG formulation provides superior sustained-release performance, leveraging the colloidal carriers to achieve prolonged and controlled drug delivery, offering a promising strategy for prolonged ocular drug delivery.

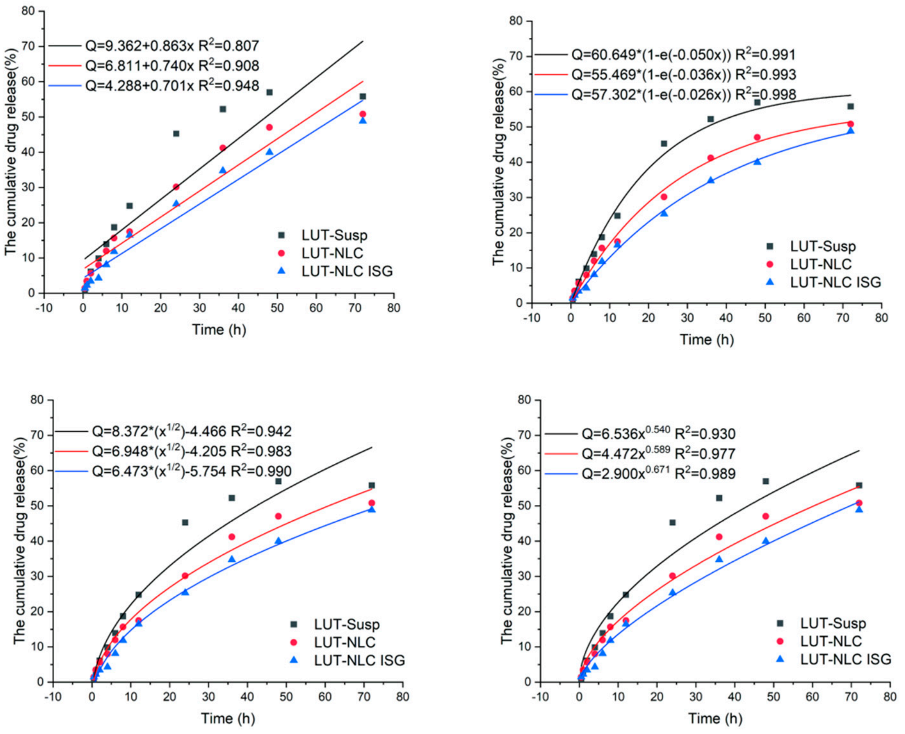

Figure S4 Kinetic models fitted to the drug release data: (A) Zero order, (B) First order, (C) Higuchi, (D) Korsmeyer-Peppas.

Section S5 Morphology observation of blank NLC and LUT-NLC

Transmission electron microscopy (TEM, FEI200C, Thermo Fisher, Waltham, MA, USA) was used to observe the morphology of blank NLC and LUT-NLC. A drop of each formulation was placed onto a separate copper grid. After removing the excess solution with filter paper, the grids were negatively stained with 2% (w/v) uranyl acetate for 1 min. Once dried, the copper grids were loaded into the TEM system for imaging. The average nanoparticle size was determined by analyzing the TEM images using ImageJ software (version 1.41, NIH, USA).

TEM images showed spherical, uniform droplets for LUT-NLC (Figure S5), consistent with the DS distribution. Measurements showed that the particle size of LUT-NLC was about 5 nm larger than that of the blank NLC, indicating that the drug was successfully encapsulated by NLC.

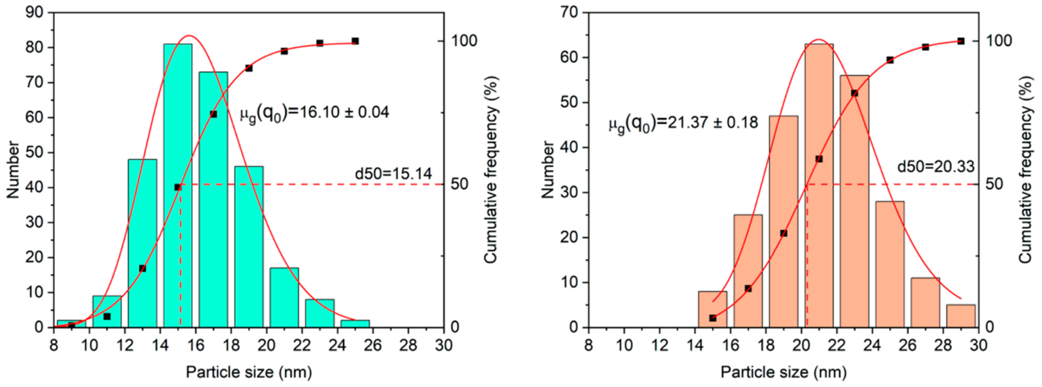

Figure S5 Histogram of particle size distribution of (A) blank-NLC; (B) LUT-NLC

Reference

1. Shi, J.; Yang, J.; Xu, H.; Luo, Q.; Sun, J.; Zhang, Y.; Liang, Z.; Zhao, N.; Zhang, J. Preparation of a Sunitinib Loaded Microemulsion for Ocular Delivery and Evaluation for the Treatment of Corneal Neovascularization in Vitro and in Vivo. *Front Pharmacol* **2023**, *14*, 1157084, doi:10.3389/fphar.2023.1157084.
2. Alsaidan, O.A.; Zafar, A.; Yasir, M.; Alzarea, S.I.; Alqinyah, M.; Khalid, M. Development of Ciprofloxacin-Loaded Bilosomes In-Situ Gel for Ocular Delivery: Optimization, In-Vitro Characterization, Ex-Vivo Permeation, and Antimicrobial Study. *Gels* **2022**, *8*, 687, doi:10.3390/gels8110687.
3. Adısanoğlu, P.; Özgüney, I. Development and Characterization of Thermosensitive and Bioadhesive Ophthalmic Formulations Containing Flurbiprofen Solid Dispersions. *Gels* **2024**, *10*, 267, doi:10.3390/gels10040267.
4. Peira, E.; Chindamo, G.; Chirio, D.; Sapino, S.; Oliaro-Bosso, S.; Rebba, E.; Ivanchenko, P.; Gallarate, M. Assessment of In-Situ Gelling Microemulsion Systems upon Temperature and Dilution Condition for Corneal Delivery of Bevacizumab. *Pharmaceutics* **2021**, *13*, 258, doi:10.3390/pharmaceutics13020258.
5. Ranch, K.M.; Maulvi, F.A.; Naik, M.J.; Koli, A.R.; Parikh, R.K.; Shah, D.O. Optimization of a Novel *in Situ* Gel for Sustained Ocular Drug Delivery Using Box-Behnken Design: In Vitro, Ex Vivo, in Vivo and Human Studies. *Int J Pharm* **2019**, *554*, 264–275, doi:10.1016/j.ijpharm.2018.11.016.
6. Ludwig, A.; van Haeringen, N.J.; Bodelier, V.M.; Van Ooteghem, M. Relationship between Precorneal Retention of Viscous Eye Drops and Tear Fluid Composition. *Int Ophthalmol* **1992**, *16*, 23–26, doi:10.1007/BF00917068.
7. Destruel, P.-L.; Zeng, N.; Maury, M.; Mignet, N.; Boudy, V. In Vitro and in Vivo Evaluation of *in Situ* Gelling Systems for Sustained Topical Ophthalmic Delivery: State of the Art and Beyond. *Drug Discov Today* **2017**, *22*, 638–651, doi:10.1016/j.drudis.2016.12.008.
8. Schroeder, S.L.M. Eyring-Polanyi Rate Theory for the Homogeneous Nucleation of Organic Crystals from Solution. *Cryst Growth Des* **2026**, *26*, 19–31, doi:10.1021/acs.cgd.5c00524.
9. Civir, G.O.; Bahadori, F.; Ozay, O.; Ergin Kızılçay, G.; Atesoglu, S.; Aldogan, E.H.; Celik, B. Design and In Vitro Evaluation of Cross-Linked Poly(HEMA)-Pectin Nano-Composites for Targeted Delivery of Potassium Channel Blockers in Cancer Therapy. *Gels* **2025**, *12*, 13, doi:10.3390/gels12010013.
